# Treatment for Stable Coronary Artery Disease: A Network Meta-Analysis of Cost-Effectiveness Studies

**DOI:** 10.1371/journal.pone.0098371

**Published:** 2014-06-04

**Authors:** Thibaut Caruba, Sandrine Katsahian, Catherine Schramm, Anaïs Charles Nelson, Pierre Durieux, Dominique Bégué, Yves Juillière, Olivier Dubourg, Nicolas Danchin, Brigitte Sabatier

**Affiliations:** 1 Pharmacie, Hôpital Européen Georges Pompidou, APHP, Paris, France; 2 URC Hôpital Henri Mondor, APHP, Créteil, France; 3 Equipe 22, Centre de Recherche des Cordeliers, UMRS 762 INSERM, Paris, France; 4 Département de Santé Publique et Informatique, Hôpital Européen Georges Pompidou, APHP, Paris, France; 5 Faculté de Pharmacie, Université René Descartes, Paris, France; 6 Cardiologie, Institut Lorrain du Cœur et des Vaisseaux Louis Mathieu, Nancy, France; 7 Cardiologie, Hôpital Ambroise Paré, APHP, Boulogne Billancourt, France; 8 Université de Versailles-Saint Quentin, Montigny-Le-Bretonneux, France; 9 Cardiologie, Hôpital Européen Georges Pompidou, APHP, Paris, France; 10 Faculté de Médecine, Université René Descartes, Paris, France; National Taiwan University, Taiwan

## Abstract

**Introduction and Objectives:**

Numerous studies have assessed cost-effectiveness of different treatment modalities for stable angina. Direct comparisons, however, are uncommon. We therefore set out to compare the efficacy and mean cost per patient after 1 and 3 years of follow-up, of the following treatments as assessed in randomized controlled trials (RCT): medical therapy (MT), percutaneous coronary intervention (PCI) without stent (PTCA), with bare-metal stent (BMS), with drug-eluting stent (DES), and elective coronary artery bypass graft (CABG).

**Methods:**

RCT comparing at least two of the five treatments and reporting clinical and cost data were identified by a systematic search. Clinical end-points were mortality and myocardial infarction (MI). The costs described in the different trials were standardized and expressed in US $ 2008, based on purchasing power parity. A network meta-analysis was used to compare costs.

**Results:**

Fifteen RCT were selected. Mortality and MI rates were similar in the five treatment groups both for 1-year and 3-year follow-up. Weighted cost per patient however differed markedly for the five treatment modalities, at both one year and three years (P<0.0001). MT was the least expensive treatment modality: US $3069 and 13 864 after one and three years of follow-up, while CABG was the most costly: US $27 003 and 28 670 after one and three years. PCI, whether with plain balloon, BMS or DES came in between, but was closer to the costs of CABG.

**Conclusions:**

Appreciable savings in health expenditures can be achieved by using MT in the management of patients with stable angina.

## Introduction

Expenses related to the management of coronary artery disease represent a considerable burden for healthcare systems. The estimated direct and indirect cost of heart disease in 2010 in the USA was $177.13 billion [1]. The recent increase in expenditure can be explained by the rising number of invasive procedures, and by higher costs for percutaneous coronary intervention (PCI) due to the widespread use of drug-eluting stents (DES). In the USA, coronary revascularization is one of the most common major medical interventions provided by the healthcare system; between 2001 and 2008, the number of coronary revascularization procedures rose by 6% with over 1 million performed in 2006 [2]. In the same year, in the USA, over 70% of PCI were performed with DES [3]. Although DES do reduce the risk of repeat procedures as compared to bare-metal stents (BMS), widespread use of this technique has not led to the anticipated reduction in the total number of procedures performed [4].

Clinical data have failed to demonstrate clear superiority of any of the treatment modalities available (medical therapy alone, PCI or coronary artery bypass graft [CABG]) for stable coronary artery disease in terms of hard clinical events [5]–[7] for non-specific populations [8] (i.e., patients with diabetes, peripheral arterial disease, etc). Comparing the costs of these different management strategies therefore appears warranted and numerous studies have previously assessed the cost-effectiveness of the different pairwise therapeutic options [9]–[14].

In order to clarify this important public healthcare issue, we set out to compare, through a network meta-analysis, the efficacy and mean cost per patient (after one and three years of follow-up) of the following treatments as assessed in randomized controlled trials (RCT): MT, percutaneous coronary intervention without stent (PTCA), with BMS, with -DES, and elective CABG.

## Methods

### Search strategy

Our strategy involved searching Medline via PubMed, Embase and the Cochrane library and relevant websites (www.theheart.org, www.pcronline.com, www.tctmd.com, www.clinicaltrialresults.org, www.crtonline.org and stent manufacturer web pages). The search was also extended to proceedings of the American Heart Association, the American College of Cardiology, the British Cardiac Society and the European Society of Cardiology.

Keywords (used as free text words) for the PubMed search were "coronary artery disease", "myocardial revascularization" and "costs". We selected the following filters: "humans", "clinical trial" (for the type of study) and "English" for the language. The same keywords were used to search in the Cochrane Library. For the Embase search, two keywords were combined: "ischemic heart disease" and "cost". The search was filtered on the term "humans", and limited to RCT. Lastly, to avoid duplication, we excluded the PubMed database that is accessible via Embase (figure S1).

The search was restricted to the period between January 1, 1980 and June 1, 2012.

Two authors (TC and BS) independently reviewed titles, abstract, and the full text as required to determine whether the studies met our inclusion criteria. Any conflict between reviewers was resolved by re-review and discussion.

### Inclusion and exclusion criteria

We conducted our analysis on adult patients with stable or stabilized unstable angina (stabilization was defined by symptoms older than 48 hours) or documented silent myocardial ischemia. All patients were assessed, regardless of whether they had single or multivessel coronary artery disease. Indeed, for these clinical situations, all three treatment modalities (MT only, PCI, CABG) are considered possible alternatives by learned societies, based on the results of RCT and the related meta-analyses [5]–[7], [15], [16]. We did not include RCT where patients had acute coronary syndromes (ACS), non-stabilized unstable angina (symptoms within 48 hours of randomization) or myocardial infarction in the previous 48 hours (MI), because revascularisation is usually considered the preferred therapeutic option for such patients [17], [18]. We also excluded studies performed in patients with in-stent restenosis because revascularization is the standard approach in these situations [19]–[21].

We selected all published randomized controlled trials that documented at least two of the five treatment modalities: MT, PTCA without stent, PCI with BMS or DES and elective CABG with cardiopulmonary bypass. Time periods with at least one event in any group were included in the analyses.

Studies were included in the clinical review if they reported 1) rates of death and MI, and 2) direct costs due to medical expenses for the management of the disease over a follow-up period of one year and/or three years. Indeed, costs relating to treatment of stable coronary artery disease are related to hospitalization due to complications such as subsequent revascularisation, MI and death.

We excluded all studies focusing on specific patient profiles, such as those with as diabetes mellitus, all studies with data based on economic models, and studies on non-conventional treatment modalities such as off-pump coronary artery bypass grafting, complete vessel treatment with PCI, etc. Lastly, all studies comprising clinical data alone were also excluded.

### Data extraction and cost conversions

We recorded information from each trial about the publication (first author, journal, and year of publication); patient demographics (mean age, proportion of men, prior revascularization, prior MI, diabetic participants, and patients with multivessel disease); the type of treatments that were compared and the number of patients assigned to each group; years of patient enrolment; whether the trial was blinded; and follow-up duration.

We recorded death and MI rates in each arm of the studies. To study the economical outcome we sought the direct costs related to treatment in each study. We extracted the direct medical-care costs for the management of the disease. Costs were recorded with the currency and year of calculation.

A cascading adjustment was made to generate costs for the patient that would be comparable across the different countries. We used a comparison adjustment by purchasing power parity (PPP). The costs recorded ineach study were converted into US $ 2008 and then: 1) costs were collected in the original currency used in the study; 2) if necessary, costs were converted into the currency of the country where the economic study was conducted; 3) between the year the costs were calculated and 2008, we applied the consumer price index of the country where the economic study was conducted; 4) costs were converted to US $ 2008 using the PPP in 2008 (available on the Organisation for Economic Co-operation and Development website [15]). The currency conversion rate expresses the purchasing power of different currencies in one common unit (i.e. US $); it incorporates not only the exchange rates between currencies, but also the amount of currency needed to buy the same basket of goods and services in different countries. This method has been used previously in several studies [16]–[18].

Additionally, for each RCT, we recorded the source of the costs studied. Direct costs of treatment for stable coronary artery disease were related to hospitalisation (for an initial revascularization procedure or for management of complications), to outpatient care (medical visit, radiological and biological examinations, etc), and to outpatient drug prescriptions (antiplatelet drugs, antianginal drugs, etc).

### Assessment of methodological quality

Quality was evaluated using two checklists. Relevance of clinical data was assessed using methods put forward by the Heart Collaborative Review Group [19]. The 4 criteria considered are: the randomization process, the allocation concealment process, the potential for selection bias after allocation and the adequacy of masking. For each criterion, three or four answers are possible, "A" being the best. The “Drummond checklist” was used to measure the methodological quality of full economic evaluations conducted alongside single effectiveness studies [20]. This checklist evaluates 35 criteria grouped into three themes: study design, data collection, and analysis and interpretation of results. These checklists are presented in the figures S2 and S3.

### Statistical analysis

We performed a network meta-analysis to compare MT *versus* PTCA *versus* PCI with BMS *versus* PCI and with DES *versus* CABG, separately all with regard to rates for death and MI.

Initially, Bayesian random effects models were used for multiple treatment comparisons; this approach preserves the within-trial randomised treatment comparison of each analysis. We compared the five treatments after one year of follow-up and then after three years of follow-up. Then, we used an extension of this model to compare the five treatment approaches throughout the whole follow-up period [21]–[24]. We used a random walk model based on piece-wise constant hazards to account for varying follow-up times [25]. In a random walk model, log hazard at time t depends on the log hazard at previous times. The model included random effects of the trials, adjacent time periods, interaction between trials and periods and treatment comparisons, and was fitted to the three pre-specified time periods (years 1 to 3).

Lastly, a sensitivity analysis was conducted to verify the robustness of the results. This focused only on studies that included outpatient costs (outpatient care and/or outpatient drugs) in addition to hospital costs.

Hazard ratios (HR) and cumulative incidences were estimated from the median of the posterior distribution. A HR lower than 1 indicates a benefit from the treatment. All results are given with 95% credibility intervals (CI) from the 2.5^th^ and 97.5^th^ percentiles of the posterior distribution. A result was considered significant when the CI of the HR did not contain 1. We also calculated the probability that each treatment was the best.

All results are based on 130 000 simulations with 30 000 burn-in. In all analyses, MT was considered as the reference treatment.

Mean costs, weighted by the number of patients in each study for each treatment, were calculated and compared by an ANOVA after 1 and 3 years of follow-up.

All analyses were carried out with WinBUGS version 1.4 and R version 2.12.

## Results

**Figure 1:**
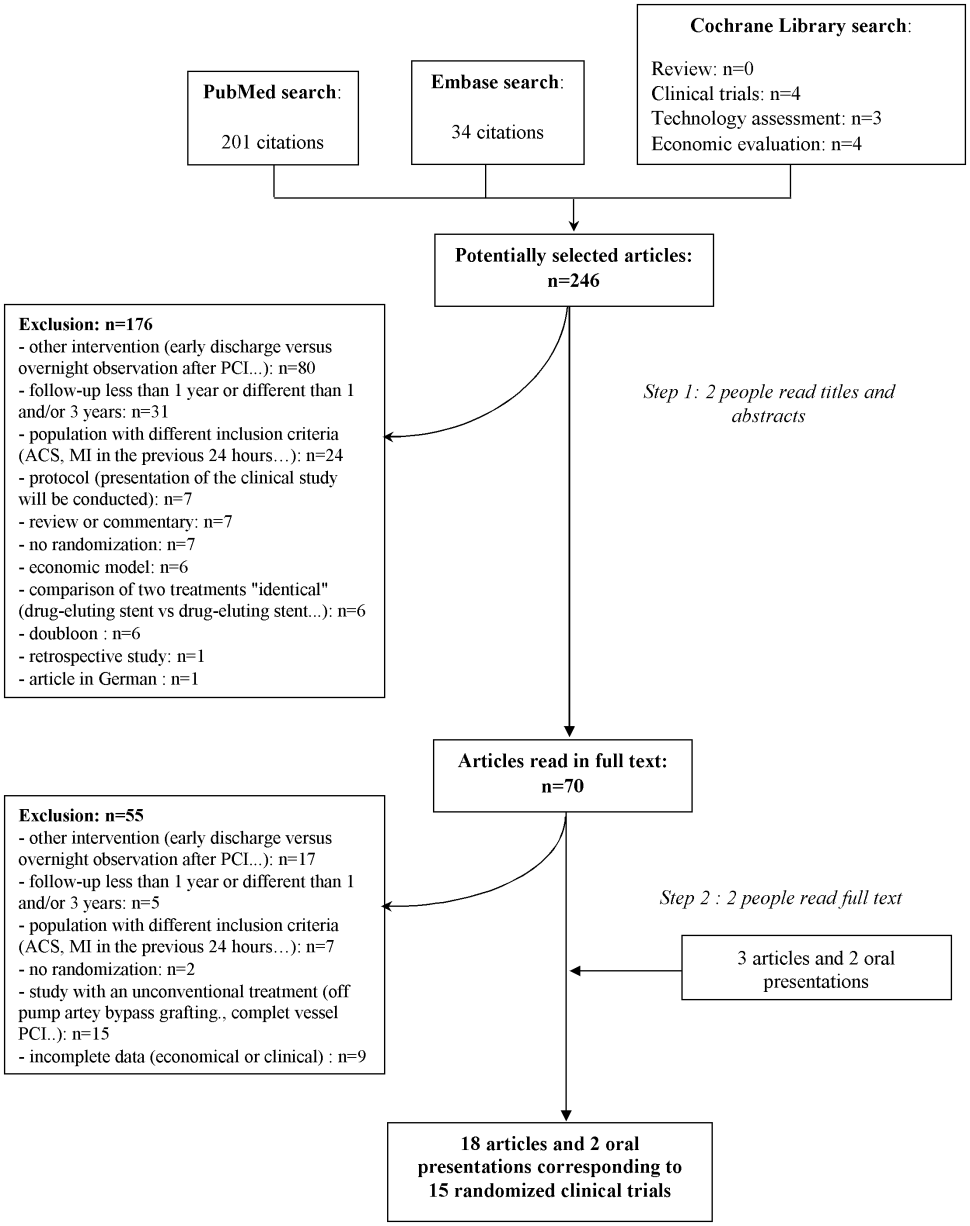
Flow diagram of the screening process.

We screened the titles and abstracts of 246 potentially eligible reports and examined the full text of 70 articles. We identified 15 RCT with 18 articles and two oral communications presented at major medical congresses that met our inclusion criteria (Figure 1): ACME [26] (*The Veterans Affairs Cooperative Study: Angioplasty Compared to Medicine*), ARTS [27], [28] (*Arterial Revascularization Therapy Study*), BENESTENT II [29] (*Randomised comparison of implantation of heparin-coated stents with balloon angioplasty in selected patients with coronary artery disease*), COURAGE [14] (*Optimal Medical Therapy with or without PCI for Stable Coronary Disease*), EAST [30] (*Emory Angioplasty Versus Surgery Trial*), ENDEAVOR II [12], [31]–[33] (*Randomized Controlled Trial to Evaluate the Safety and Efficacy of the Medtronic AVE ABT-578 Eluting Driver Coronary Stent in De Novo Native Coronary Artery Lesions*), ERACI [34], [35] (*Argentine Randomized Trial of Percutaneous Transluminal Coronary Angioplasty Versus Coronary Artery Bypass Surgery in Multivessel Disease)*, MASS II [36] (*The Medicine, Angioplasty, or Surgery Study II*), RAVEL [37] (*randomised study with the sirolimus eluting Bx Velocity balloon expandable stent in the treatment of patients with de novo native coronary artery lesions*), RITA 2 [38] (*The second Randomised Intervention Treatment of Angina*), SIRIUS [11] (*Sirolimus-Eluting Stent in De-Novo Native Coronary Lesions),* SoS [10] *(the Stent or Surgery trial*), STRESS [39] (*Stent Restenosis Study),* SYNTAX [40] *(Synergy between PCI with Taxus and Cardiac Surgery*) and TAXUS IV [41] (*A polymer-based, paclitaxel-eluting stent in patients with coronary artery disease*). The table S4 entitled “List of excluded and selected studies” presents the main reasons for exclusion.

The characteristics of included RCT are presented in table 1. Six of these trials involved patients with multivessel disease: ARTS [27], [28], COURAGE [14], EAST [30], ERACI [34], [35], MASS II [36] and SoS [10]. The other clinical trials included patients with single vessel disease. For eight trials, the duration of follow-up was one year: BENESTENT II [29], MASS II [36], RAVEL [37], SIRIUS [11], SoS [10], STRESS [39], SYNTAX [40] and TAXUS IV [41]. For four trials, 3-year follow-up was available: ACME [26], COURAGE [14], EAST [30] and RITA 2 [38]. Two trials included both 1 and 3-year follow-up data: ARTS [27], [28] and ERACI [34], [35]. Lastly, in ENDEAVOR II [12], [31]–[33], duration of follow-up was 1, 2, 3 and 4 years.

**Table 1:** Baseline characteristics of patients in the studies selected.

Study	Inclu sion	Inclusion criteria	Blinded	Single- or multi- vessel disease	Number of centres	Number of patients	Mean age (years)	Diabetes mellitus (%)	Sex (% men)	Previous MI (%)	Previous revascularisation (%)	Follow-up (years)
***PTCA versus MT***												
ACME [26]	1987–1990	stable angina pectoris, a strikingly positive exercise tolerance test or an MI within the past 3 months	no	single	8	200	62	18	100	30	0	3
RITA 2 [38]	1992–1996	angiographically-documented coronary artery disease	no	single	20	1 018	58	9	82	47	0	3
***PTCA versus CABG***												
ERACI [34], [35]	1988–1990	severe stenosis >70% in ≥1 major epicardial coronary artery, severely limiting stable angina or refractory resting unstable angina despite optimal medical therapy, no or minimal symptoms but with a large area of myocardium at risk identified by exercise testing	no	multi	1	127	57	11	85	50	NA	1 and 3
EAST [30]	1987–1990	stable or unstable angina or objective signs of ischemia	no	multi	1	392	61	23	74	41	0	3
***CABG versus MT***												
MASS II [36]	1995–2000	symptomatic multivessel coronary disease (2 or more epicardial coronary arteries with ≥70% narrowing),	no	multi	1	611	60	14	85	22	0	1
***CABG versus PCI with BMS***												
MASS II [36]	1995–2000	symptomatic multivessel coronary disease (≥2 epicardial coronary arteries with ≥70% narrowing),	no	multi	1	611	60	14	85	22	0	1
SoS [10]	1996–1999	symptomatic patients with multivessel coronary artery disease	no	multi	53	988	61	14	79	45	0	1
ARTS [27], [28]	1997–1998	stable angina pectoris or unstable angina pectoris or silent myocardial ischemia	no	multi	67	1 205	61	17	76	43	0	1 and 3
***CABG versus PCI with DES***												
SYNTAX [40]	2005–2007	stable or unstable angina pectoris with ischemia; or patients with atypical chest pain or asymptomatic with demonstrated myocardial ischemia	no	multi	85	1 740	65	28	78	32	0	1
***PCI with BMS versus PCI with DES***												
SIRIUS [11]	2001	history of stable or unstable angina and signs of myocardial ischemia.	double	single	53	1 058	62	26	71	31	NA	1
TAXUS IV [41]	2002	stable or unstable angina or inducible ischemia	double	single	73	1 314	63	24	72	30	NA	1
RAVEL [37]	2000–2001	stable or unstable angina or silent ischemia	double	single	19	238	61	19	76	36	NA	1
ENDEA VOR II [12], [31]–[33]	2003–2004	patients with clinical evidence of ischemia or a positive functional test	double	single	72	1 197	62	20	76	41	20	1, 2 and 3
***PCI with BMS versus PTCA***												
STRESS [39]	1991–1993	symptomatic ischemic heart disease	no	single	8	207	61	14	72	35	6	1
***MT versus PCI with BMS***												
MASS II [36]	1995–2000	symptomatic multivessel coronary disease	no	multi	1	611	60	14	85	22	0	1
COURAGE [14]	1999–2004	chronic angina pectoris CCS I-III, stable post-MI patients, and asymptomatic patients with objective evidence of myocardial ischemia.	no	multi	50	2 287	62	33	85	38	25	3
***CABG versus PCI with DES***												
BENE STENT II [29]	1995–1996	stable angina or unstable angina	no	single	50	823	54	12	78	26	9	1

MT: medical therapy, PTCA: percutaneous coronary angioplasty, CABG: coronary artery bypass graft, DES: drug-eluting stent, BMS: bare-metal stent, NA: not available.

**Figure 2:**
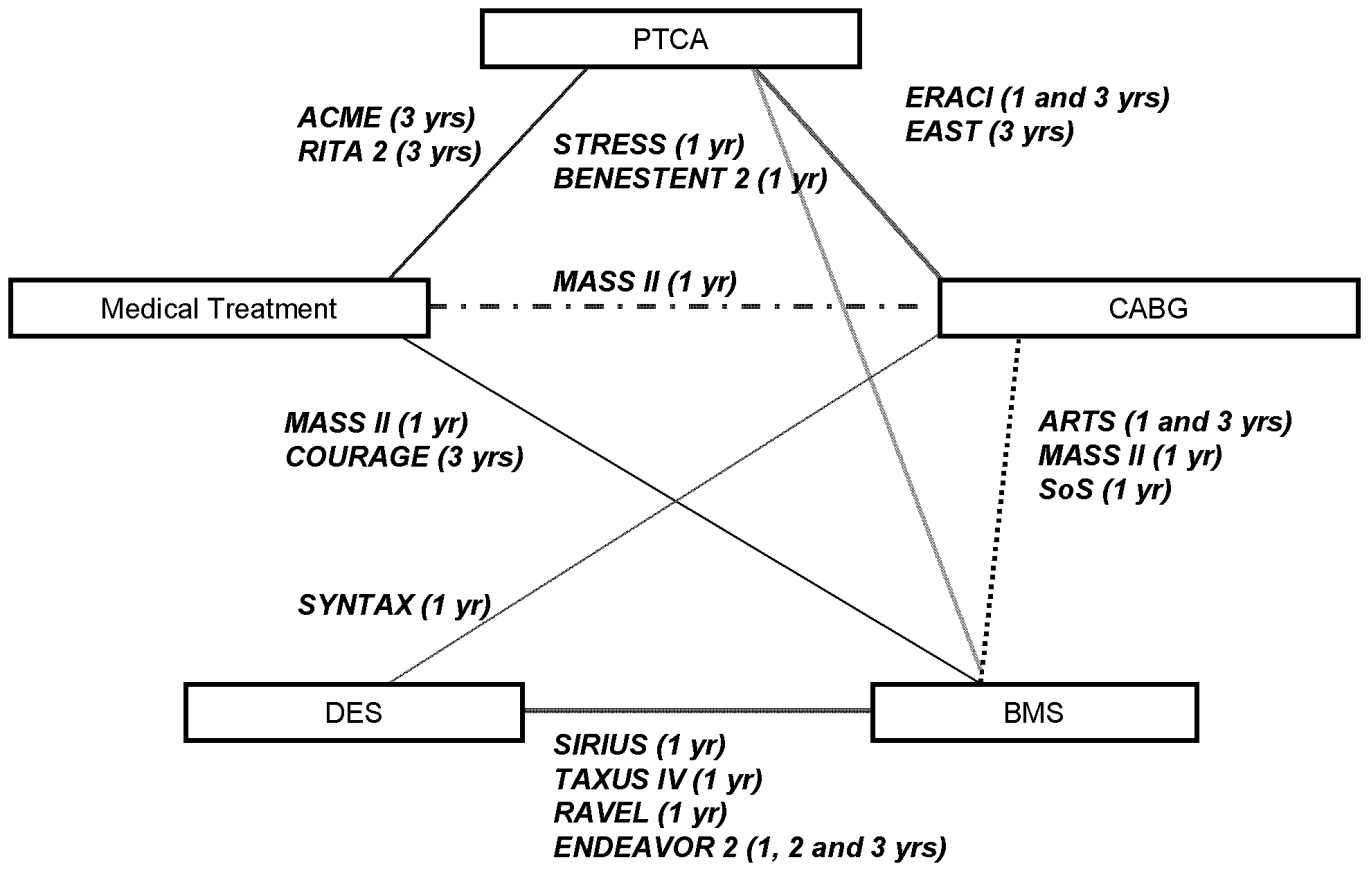
Comparators and duration of patient follow-up for the trials selected.

Figure 2 shows the comparators and the duration of patient follow-up for each RCT. The BENESTENT II [29] trial was a comparison of a heparin-coated stent *versus* PTCA. We considered this particular stent as a bare-metal stent because drug-eluting stent referred to stents with antiproliferative coating. Only one trial compared three treatment modalities: MT, BMS and CABG: MASS II [36].

According to the recommendations put forward by the Heart Collaborative Review Group, eleven trials described appropriate methods of randomization: ARTS [27], [28], BENESTENT II [29], COURAGE [14], ENDEAVOR II, MASS II [36], RAVEL [37], SIRIUS [11], SoS [10], STRESS [39], SYNTAX [40] and TAXUS IV [41]. The methods used to conceal treatment allocation were considered adequate in ten trials: ARTS [27], [28], BENESTENT II [29], COURAGE [14], MASS II [36], RAVEL [37], SIRIUS [11], SoS [10], STRESS [39], SYNTAX [40] and TAXUS IV [41]. Four of the sixteen trials were double blind: ENDEAVOR II, RAVEL [37], SIRIUS [11] and TAXUS IV [41]. Quality assessment of the clinical methodology is reported in the table S1.

According to the Drummond checklist, one trial did not specify the method used for estimating quantities and unit cost (checklist item 17): ERACI [34], [35]. Three of the eight trials with a 3-year follow-up did not apply the discount rate as recommended (checklist item 23): ARTS [27], [28],, EAST [30] and ERACI [34], [35]. For eight trials, there was no approach to sensitivity analysis (checklist item 27): ARTS [27], [28], BENESTENT II [29], EAST [30], ENDEAVOR II [12], [31]–[33], ERACI [34], [35], MASS II [36], SoS [10] and STRESS [39]. Results of quality assessment of economical methodology are displayed in the table S2.

### Clinical analysis

In all, the 15 trials included had enrolled 9 565 patients followed for one year and 6 443 patients for three years. The percentages of men or diabetic patients were similarly distributed among the treatment arms, regardless of duration of follow-up (P = 0.22 for the percentage of men and 0.23 for the percentage of diabetes mellitus).

After one year of follow-up, 202 patients died: three of the 203 patients on MT (1.5%), 60 of the 2 221 patients with CABG (2.7%), nine of the 578 patients with PTCA (1.6%), 60 of the 3 693 patients with BMS (1.6%) and 70 of the 2 796 patients with DES (2.5%). After three years of follow-up, 345 patients had died: 111 of the 1 759 patients on MT (6.3%), 43 of the 863 patients with CABG (5.0%), 39 of the 870 patients with PTCA (4.5%), 133 of the 2 336 patients with BMS (5.7%) and 19 of the 584 patients with DES (3.2%).

After one year of follow-up, 394 patients had a MI: eight of the 203 patients on MT (3.9%), 96 of the 2 221 patients with CABG (4.3%), 33 of the 578 patients with PTCA (5.7%), 171 of the 3 693 patients with BMS (4.6%) and 86 of the 2 769 patients with DES (3.1%). After three years of follow-up, 530 patients had a MI: 147 of the 1 759 patients on MT (8.3%), 80 of the 841 patients with CABG (9.5%), 67 of the 845 patients with PTCA (7.9%), 212 of the 2 336 patients with BMS (9.1%) and 19 of the 584 patients with DES (3.3%).

**Figure 3:**
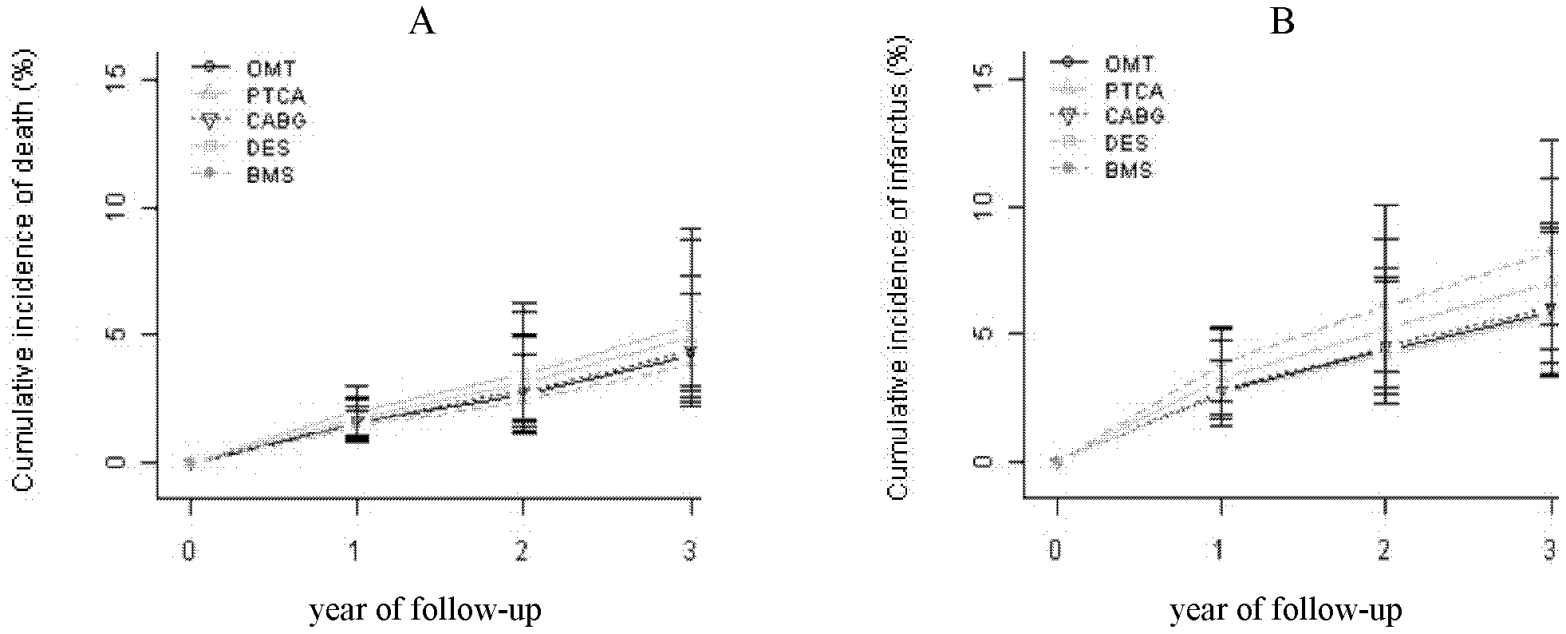
Cumulative incidences of death and MI.

After one and three years of follow-up there was no statistically significant difference between the death and MI rates of the five treatments. Because of non-significant results, the rating of treatment efficacy is not informative (Table 2 and figure 3).

**Table 2:** Comparison of the rates of death and myocardial infarction between the 5 treatments (MT *versus* CABG *versus* PTCA *versus* BMS *versus* DES).

		Events				
		MT*	CABG	PTCA	BMS	DES
**death within the first year of follow-up**	HR (95% CI)	1	2.61 (0.63; 12.55)	3.78 (0.66; 25.28)	3.10 (0.76; 15.18)	4.01 (0.95; 21.12)
	probability treatment is the best	0.87	0.06	0.05	0.02	0.00
**death within the first three years of follow-up**	HR (95% CI)	1	1.01 (0.41; 2.25)	1.24 (0.57; 2.61)	0.83 (0.41; 1.46)	0.79 (0.23; 2.56)
	probability treatment is the best	0.11	0.15	0.05	0.24	0.49
**MI within the first year of follow-up**	HR (95% CI)	1	1.07 (0.37; 2.89)	1.67 (0.47; 5.47)	1.70 (0.59; 4.57)	1.14 (0.33; 3.25)
	probability treatment is the best	0.51	0.27	0.04	0.00	0.18
**MI within the first three years of follow-up**	HR (95% CI)	1	1.48 (0.52; 5.20)	1.36 (0.57; 3.97)	1.76 (0.72; 3.45)	1.03 (0.23; 6.11)
	probability treatment is the best	0.37	0.07	0.09	0.02	0.45

HR: hazard ratio, MI: myocardial infarction, MT: medical therapy, PTCA: percutaneous coronary angioplasty, CABG: coronary artery bypass graft, DES: drug-eluting stent, BMS: bare-metal stent, CI: confidence interval.

* medical therapy was the reference treatment.

### Economic analysis

**Figure 4:**
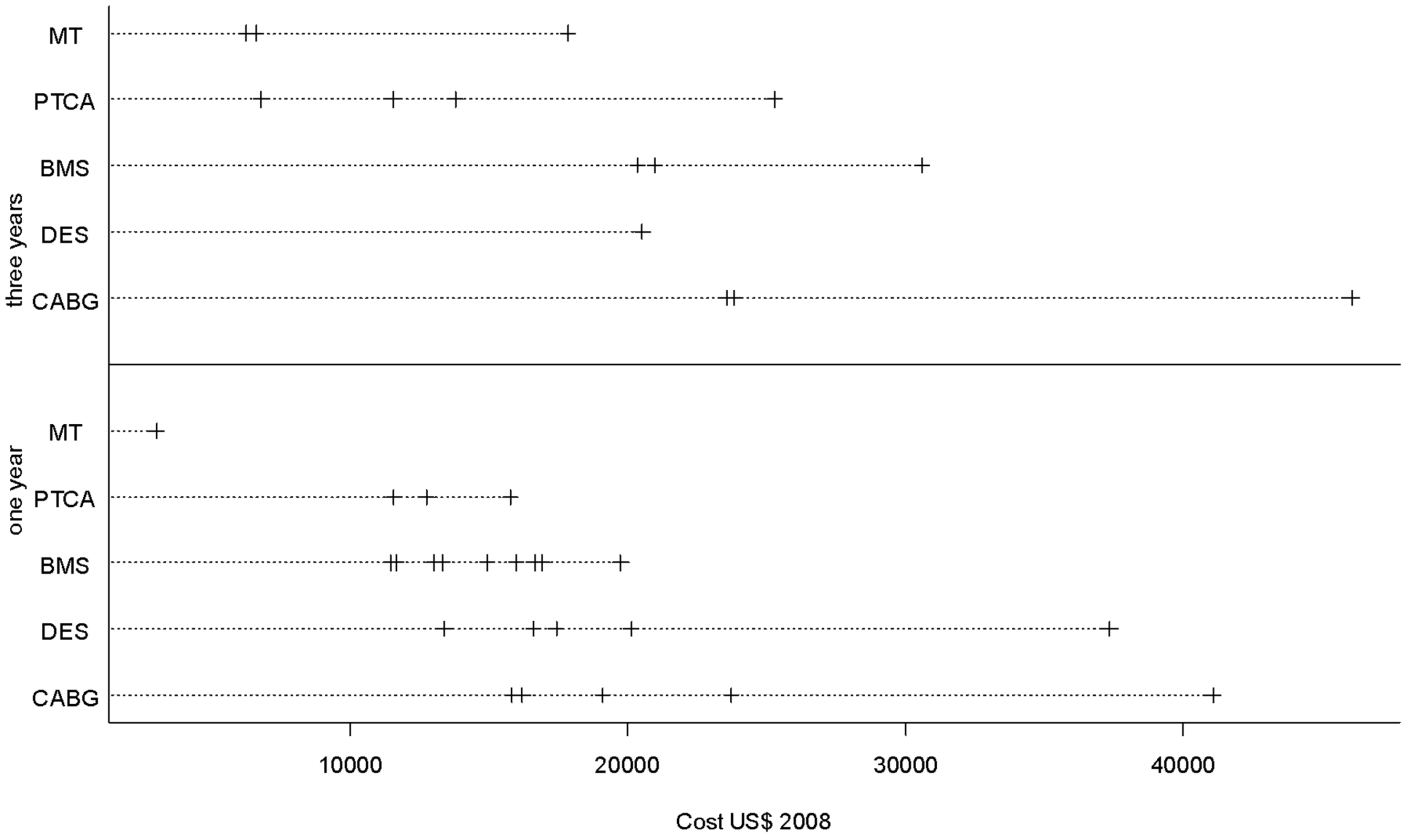
Cost per patient adjusted in US $ 2008 after 1 and 3 years of follow-up (each mark represents a clinical study).

**Figure 5:**
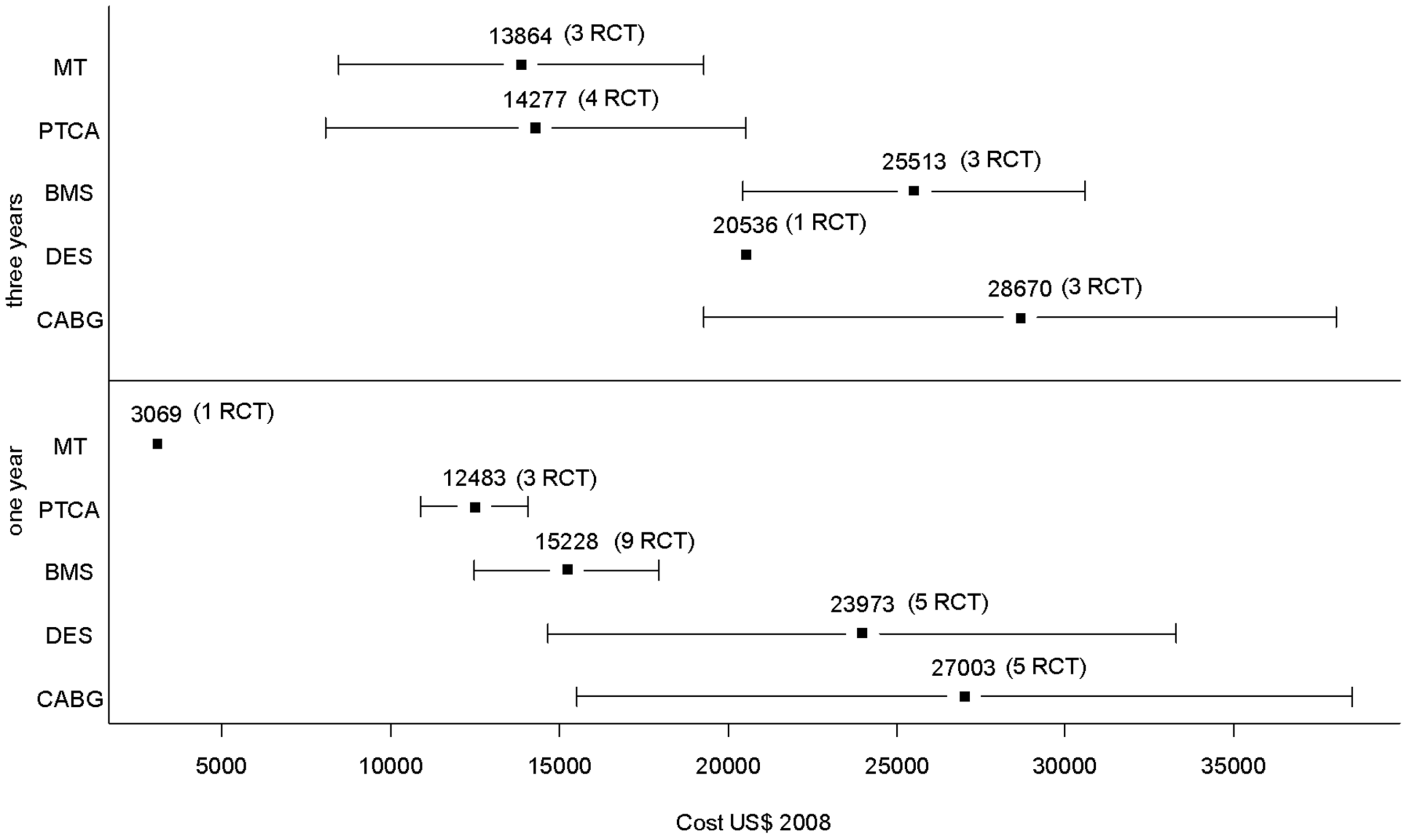
Mean weighted cost per patient in US $ 2008 and standard deviation (number of RCT available).

Table 3 and figure 4 present the evaluation of cost per patient for each RCT: cost published in the article (year of publication and currency used) and cost per patient adjusted in US $ 2008. Figure 5 presents the mean cost per patient for each treatment. After one year of follow-up, the mean weighted cost per patient in US $ 2008 was: $3069 with MT, $27 003 with CABG, $12 483 with PTCA, $15 228 with BMS, and $23 973 with DES. After three years of follow-up, the mean weighted cost was: $13 864 with MT, $28 670 with CABG, $14 277 with PTCA, $25 513 with BMS, and $20 536 with DES. There was a statistically significant difference of weighted cost per patient for the comparison of the five treatments: P value was <0.0001 after one year and after three years. Between one and three years of follow-up, the greatest increase in average weighted cost per patient was observed with MT (+ $10 795, +352% compared with the cost per patient after one year). During this period, weighted cost with treatment by CABG was stable (+ $1667, +6% compared with cost per patient after one year). We performed a comparison of the weighted cost of each treatment in at least two clinical studies. For all these comparisons, at one and three years of follow-up, the differences observed were significant (P<0.0001): CABG versus PTCA after one year, CABG versus BMS after one year, CABG versus DES after one year, etc.

**Table 3:** Cost per patient for each treatment.

	Trial	FU (year)	Cost per patient as published (currency, year, country)	Cost followed	multi or single vessel disease	Cost per patient adjusted (US $ 2008)
**MT**	MASS II	1	2 285 (US $, 1998, Netherlands)	H+D	MVD	3 069
	ACME	3	6 311 (Aus $, 1994, Australia)	H+C+D	SVD	6 299
	RITA 2	3	3 613 (£, 1999, UK)	H+C+D	SVD	6 633
	COURAGE	3	15 653 (US $, 2004, USA)	H+C+D	MVD	17 842
**CABG**	ARTS	1	13 638 (US $, 1998, Netherlands)	H+C	MVD	19 100
	ERACI	1	12 938 (US $, 1991, Argentina)	H	MVD	23 733
	MASS II	1	11 794 (US $, 1998, Netherlands)	H+D	MVD	15 846
	SoS	1	8 905 (£2000, UK)	H+D	MVD	16 222
	SYNTAX	1	39 581 (US $, 2007, USA)	H+C+D	MVD	41 101
	ARTS	3	16 100 (€, 1998, Netherlands)	H+C	MVD	23 596
	EAST	3	25 310 (US $, 1997, USA)	H	MVD	46 083
	ERACI	3	13 000 (US $, 1991, Argentina)	H	MVD	23 847
**PTCA**	BENESTENT II	1	16 727 (Dfl, 1996, Netherlands)	H	SVD	11 596
	STRESS	1	10 865 (US $, 1994, USA)	H	SVD	15 782
	ERACI	1	6 952 (US $, 1991, Argentina)	H	MVD	12 753
	ACME	3	6 790 (Aus $, 1994, Australia)	H+C+D	SVD	6 777
	RITA 2	3	6 299 (£1999, UK)	H+C+D	SVD	11 565
	ERACI	3	7 523 (US $, 1991, Argentina)	H	MVD	13 800
	EAST	3	23 734 (US $, 1997, USA)	H	MVD	25 310
**BMS**	BENESTENT II	1	18 812 (Dfl, 1996, Netherlands)	H	SVD	13 041
	ENDEAVOR II	1	16 641 (US $, 2008, USA)	H	SVD	16 641
	RAVEL	1	9 915 (€, 2001, Netherlands)	H+D	SVD	13 339
	SIRIUS	1	16 504 (US $, 2002, USA)	H+C+D	SVD	19 755
	STRESS	1	11 656 (US $, 1994, USA)	H	SVD	16 931
	TAXUS IV	1	14 011 (US $, 2004, USA)	H+D	SVD	15 971
	ARTS	1	10 665 (US $, 1998, Netherlands)	H+C	MVD	14 936
	MASS II	1	8 676 (US $, 1998, Netherlands)	H+D	MVD	11 656
	SoS	1	6 296 (£2000, UK)	H+D	MVD	11 469
	ENDEAVOR II	3	20 348 (US $, 2008, USA)	H	SVD	20 348
	ARTS	3	14 302 (€, 1998, Netherlands)	H+C	MVD	20 961
	COURAGE	3	26 847 (US $, 2004, USA)	H+C+D	MVD	30 602
**DES**	ENDEAVOR II	1	17 422 (US $, 2008, USA)	H	SVD	17 422
	RAVEL	1	9 969 (€, 2001, Netherlands)	H+D	SVD	13 412
	SIRIUS	1	16 813 (US $, 2002, USA)	H+C+D	SVD	20 124
	TAXUS IV	1	15 447 (US $, 2004, USA)	H+D	SVD	16 624
	SYNTAX	1	35 991 (US $, 2007, USA)	H+C+D	MVD	37 373
	ENDEAVOR II	3	20 536 (US $, 2008, USA)	H	SVD	20 536

FU: follow-up, MT: medical therapy, PTCA: percutaneous coronary angioplasty, CABG: coronary artery bypass graft, DES: drug-eluting stent, BMS: bare-metal stent, SVD: single vessel disease, MVD: multi vessel disease, H: hospital cost, C: costs related to outpatient care, D: costs related to outpatient drugs.

For five trials, only hospital costs were assessed: BENESTENT II [29], ENDEAVOR II [12], [31]–[33], EAST [30], ERACI [34], [35] and STRESS [39]. For ARTS [27], [28], costs related to outpatient medical visits were studied in addition to hospital costs. In four studies, the costs assessed were related to hospitalization and outpatient drugs: MASS II [36], RAVEL [37] TAXUS IV [41] and SoS [10]. In five studies, costs assessed were related to hospitalization, outpatient care (medical visit and/or cardiovascular testing) and outpatient drugs: ACME [26], COURAGE [14], RITA 2 [38], SIRIUS [11] and SYNTAX [40].

### Sensitivity analysis

In this analysis, we excluded trials reporting only hospital costs: BENESTENT II [29], ENDEAVOR II [12], [31]–[33], EAST [30], ERACI [34], [35] and STRESS [39]. Consequently, not all treatment modalities could be compared at one year and three years of patient follow-up: at one year, data on PTCA alone were not available for the sensitivity analysis; likewise, data on DES after three years of follow-up could not be used in the sensitivity analysis.

After one year of follow-up, results of the sensitivity analysis were consistent with the main results. MT remained the least expensive, followed by BMS, then DES and lastly CABG (P<0.0001). After 3 years however, the results were different. Treatment with PTCA appeared to be the least expensive and CABG was still the most costly strategy (P<0.0001). Table S3.

## Discussion

The present network meta-analysis confirms the absence of a statistically significant difference between medical therapy, angioplasty without stent, angioplasty with BMS, angioplasty with DES and coronary artery bypass graft on mortality and myocardial infarction rates at one and three years of follow-up. These results concord with those reported in recent meta-analyses and therefore justify the cost-comparison of the different treatment strategies [5]–[7], [42].

Our economic analysis demonstrates a significant difference of weighted costs per patient between the five treatment options. Medical therapy is the least expensive with a weighted cost per patient of US $3069 after one year of follow-up and US $13 864 after three years of follow-up. Coronary artery bypass grafting is the most costly treatment modality: US $27 003 and US $28 670 at one and three years respectively. Between one and three years of follow-up, however, the largest increase in average weighted cost per patient was observed with MT (+ $10 795, +352% compared with the cost after one year), followed by BMS (+ $10 285, +67%), then PTCA (+ $1794, +14%) and CABG (+ $1667, +6%). This significant increase in expenditures, particularly for the MT group, can probably be explained by the need for (additional) revascularization during mid-term follow-up. The sensitivity analysis, performed on the 10 studies that followed, in addition to hospital costs, outpatient costs (outpatient care and/or outpatient drugs), yielded results that are consistent with those of the primary analysis after one year of follow-up. At three years, balloon PTCA and MT had comparable low costs, while there was little difference in the costs of BMS and CABG.

The apparent decrease in cost from one year to three years with DES is artefactual, and due to the fact that only one trial (ENDEAVOR II [12], [31]–[33]) reported 3-year results, whereas several trials were pooled to derive one-year costs. When considering change in costs from one to three years in ENDEAVOR II, an 18% increase was observed, which is consistent with the reduced need for additional revascularization with DES, compared with BMS [12]. The cost increase in ENDEAVOR II is in line with that found in ENDEAVOR III, a clinical trial comparing two different DES: +23% for the sirolimus-eluting stent and +24% for the zotarolimus-eluting stent [43]. In addition, after three years of follow-up we observed a lower cost per patient for the treatment with PTCA compared with treatment with BMS. This surprising observation can probably be explained by the different proportion of patients with SVD: 70% of patients treated by PTCA versus 25% in the BMS group after three years of follow-up.

Overall, the increased initial cost related to initial performance of myocardial revascularization was not counterbalanced by equivalent savings during the three subsequent years of follow-up, although the difference at one year was notably attenuated at three years. A cost advantage for MT compared with myocardial revascularisation was also observed in BARI 2D after four years of follow-up [44]. In this study, which only included patients with type 2 diabetes mellitus, medical costs per patient were higher for CABG or PCI than for MT. Costs (in US$ 2007) were 80 900, 73 400 and 65 600 respectively after four years of follow-up.

Because of the relatively small number of studies in the meta-analysis, and as we did not use individual data, we were unable to perform separate analyses for patients with single-vessel disease versus multivessel disease. It is possible that the benefit of MT in terms of costs might be more limited in patients with multi-vessel coronary artery disease who are more likely to need subsequent myocardial revascularization.

Nowadays, plain balloon angioplasty (PTCA) is only used in very rare instances. We did keep these studies in our analysis, however, in order to provide additional data on the treatment modalities studied in the non-PTCA arms of the trials (CABG or medical treatment); indeed, excluding the six studies using balloon angioplasty would have resulted in also excluding two studies with medical treatment (ACME and RITA 2), two studies with CABG (ERACI and EAST) and two studies with BMS (BENESTENT 2 and STRESS), thereby much decreasing the statistical power of our analyses.

We also adopted the approach to consider BMS and DES studies separately. In fact, although DES are increasingly used, a substantial proportion of procedures still use BMS, with wide between-country variations; the proportion of DES in published studies varied from 23% (Sweden, 2007, Gudnason et al.), 45% (France, 2004-2008, Puymirat et al.), 61% (Spain, 2009, Diaz J et al.) and 70% (USA, 2011, Dehmer GJ et al.) [45]-[48].

### Critical appraisal of costing methods

We must emphasize that the definition of costs in each study varies. In the management of coronary artery disease, direct costs correspond to three items of expenditure: hospitalization (for invasive treatment and/or care of complications of the disease), outpatient care (medical visits, radiological and biological tests, home visits by nurses, etc.) and outpatient medications (anti-platelet drugs, anti-anginal drugs, etc). All 15 studies included in our economical analysis assessed hospitalization-related costs. Among the 15 RCT, 10 measured both hospital and ambulatory costs (assessment of ambulatory care and /or medication costs). For seven of these 10 RCT, separate hospital and ambulatory cost analyses were available. After one year, ambulatory costs represented an average of 8% of the total cost (from 2.9% in TAXUS-IV to 15% in SoS); after three years, only one study (RITA-2) provided a detailed analysis of the respective proportion of hospital and ambulatory costs; as expected, the percentage of the total cost related to ambulatory care was higher than that observed at one year. Furthermore, a number of the studies analysed the cost of all cardiovascular drugs (ARTS, SYNTAX, RITA 2 etc), whereas the SIRIUS trial analysed the cost of thienopyridines only.

Moreover, the methods used to calculate the cost per patient vary in the studies analyzed. In practice, as published by Reed et al. [49], the calculation of the average cost depends on two parameters. The first is the approach used in the clinical trial to estimate the resource consumed. Indeed, resource-use can be based on data from patients in all countries participating in the clinical trial or from patients belonging to one center or one country. The second is the costing approach; again, the unit cost applied for the whole trial population can be derived from individual countries, from a single country, or from one center. According to these two parameters, studies can be classified into six groups: fully pooled with multi-country costing, fully pooled with one-country costing, partially split with multi-country costing, partially split with one-country costing, fully split with multi-country costing and fully split with one-country costing. The 15 trials we analyzed belong to two of these six groups. Eight trials are classified as “fully pooled with one-country costing”: ARTS [27], [28], BENESTENT II [29], COURAGE [14],, ENDEAVOR II [12], [31]–[33], RAVEL [37], RITA 2 [38], SoS [10] and SYNTAX [40]. The seven other studies are classified as “fully split with one-country costing”: ACME [26], EAST [30], ERACI [34], [35], MASS II [36], SIRIUS [11], STRESS [39] and TAXUS IV [41]. Our conclusions may therefore be limited by the methodological variability of the 16 trials we analyzed, although the results were fairly consistent regardless of the costing methods used.

### Limitations

Our meta-analysis included trials that were conducted at a time when the technique of PCI used would be considered completely obsolete in today's terms. In such earlier studies, the rates of subsequent revascularisation following PCI were definitely higher than those that would currently be observed, leading to higher follow-up costs than would be found nowadays.

Also, as some trials planned angiographic follow-up for all patients, including those who were asymptomatic, the rates of repeat revascularization may have been higher that those that would have been observed in a real-life situation, because of the “oculostenotic reflex” that mandatory coronary angiography during follow-up may have induced. In fact, only six studies did not include routine angiographic follow-up: ARTS [27], [28], COURAGE [14], ERACI [34], [35], RITA 2 [38], SoS [10] and SYNTAX [40]. Studies in which angiographic follow-up was planned, tended to have higher treatment costs.

Another limitation of our analysis is that the period over which we selected the studies expands over two decades, with the oldest trial (EAST) recruiting patients from 1987 to 1990, and the most recent (SYNTAX) between 2005 and 2007. During this period, the cost of BMS and DES has decreased substantially. Among the trials selected, five reported the unit cost of stents (RAVEL, SIRIUS, TAXUS IV, SYNTAX et ENDEAVOR II): the cost of BMS remained relatively stable (US $900 to 700) while the absolute price decrease for DES was greater (from US $2900 to 2100). The differential cost between BMS and DES remained stable (≈ US $2000), except for the two most recent trials (SYNTAX, 2007 and ENDEAVOR II, 2008), where the difference was smaller. Although we lacked the power to fully take these changes into consideration, they should be kept in mind when analysing our results.

In addition, it must be emphasized that the nature of costs varied across the 15 RCT. All measured hospital costs, while 10 also assessed ambulatory costs. A specific sensitivity analysis, however, was performed to take this variability into account.

Lastly, we used data from one single randomized clinical trial in two situations: medical therapy with one year follow-up (MASS II [36]) and treatment with DES with three years of follow-up (ENDEAVOR II [12], [31]–[33]). As mentioned above, this may have led to inconsistencies, such as the seemingly lower costs from one to three years in patients with DES.

## Conclusions

This network meta-analysis documents considerable differences in treatment costs at 3-year follow-up, when comparing five treatment modalities that provided similar clinical results, in terms of death and risk of myocardial infarction. Medical therapy in patients without acute coronary syndromes therefore appears to be the most cost-effective option, which may achieve appreciable savings in healthcare expenditures. Our findings, however, may be limited by methodological considerations pertaining to the way costs are evaluated in long-term randomized trials, and by the fact that we did not take into account potential differences between treatment modalities in terms of symptoms.

## Supporting Information

Figure S1 **Search strategy in Medline, Embase and Cochrane library.**
(DOC)

Figure S2 **Checklist of clinical quality assessment according to the Heart Collaborative Review Group.**
(DOC)

Figure S3 **Drummond checklist.**
(DOC)

Table S1 **Clinical quality assessment.**
(DOC)

Table S2 **Economical quality assessment.**
(DOC)

Table S3 **Sensitivity analyse for the comparison MT** ***versus*** **CABG** ***versus*** **PTCA** ***versus*** **BMS** ***versus*** **DES.**
(DOC)

Table S4 **List of selected or rejected articles and reason in case of exclusion.**
(DOC)

Checklist S1 **PRISMA checklist.**
(DOC)
