# Supplementary figures and images for: Treatment for Stable Coronary Artery Disease: A Network Meta-Analysis of Cost-Effectiveness Studies
Source: PLoS One. 2014 Jun 4;9(6):e98371. doi: 10.1371/journal.pone.0098371 (PMC4045726; doi:10.1371/journal.pone.0098371)

Figure S1: Search strategy in Medline, Embase and Cochrane library


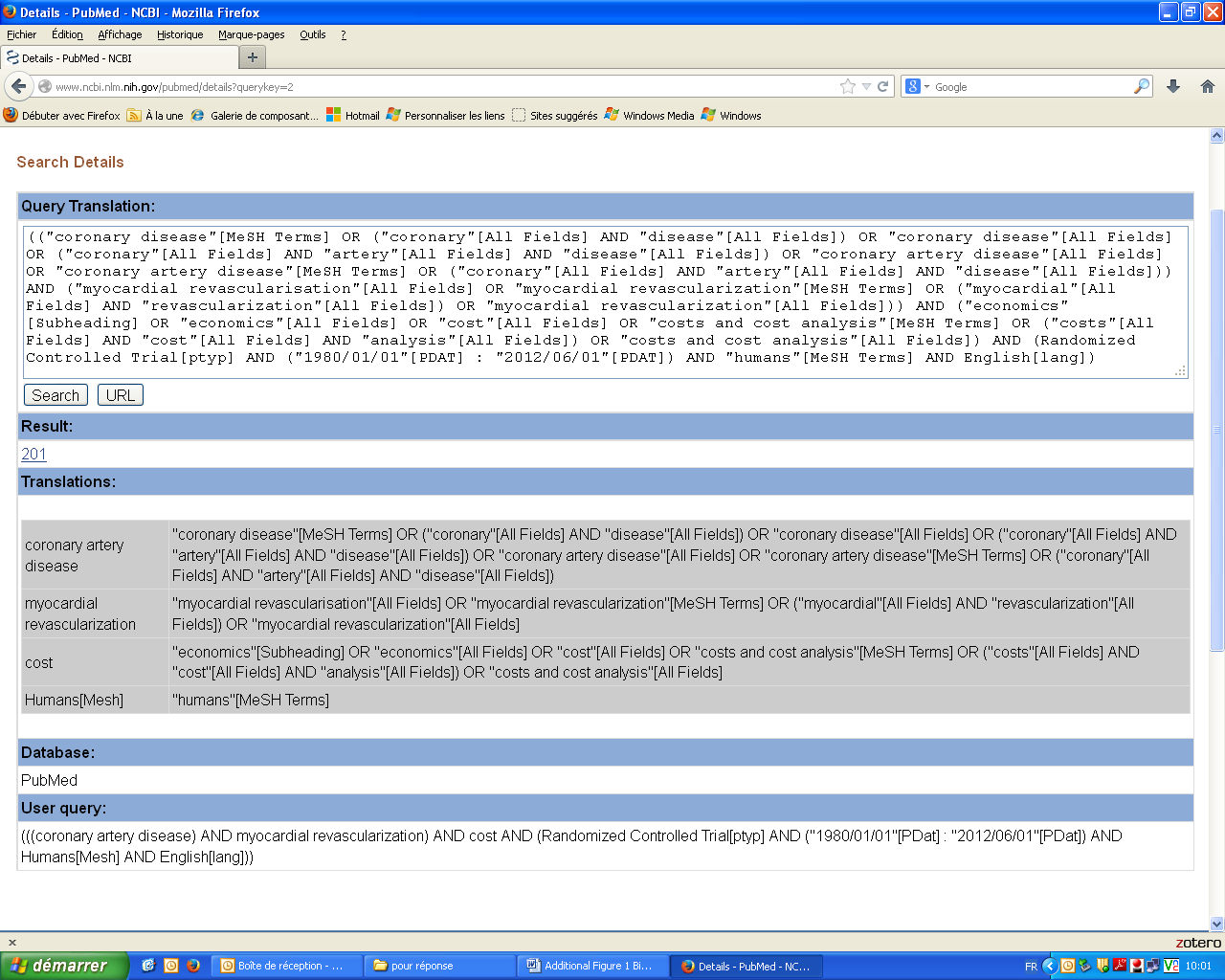


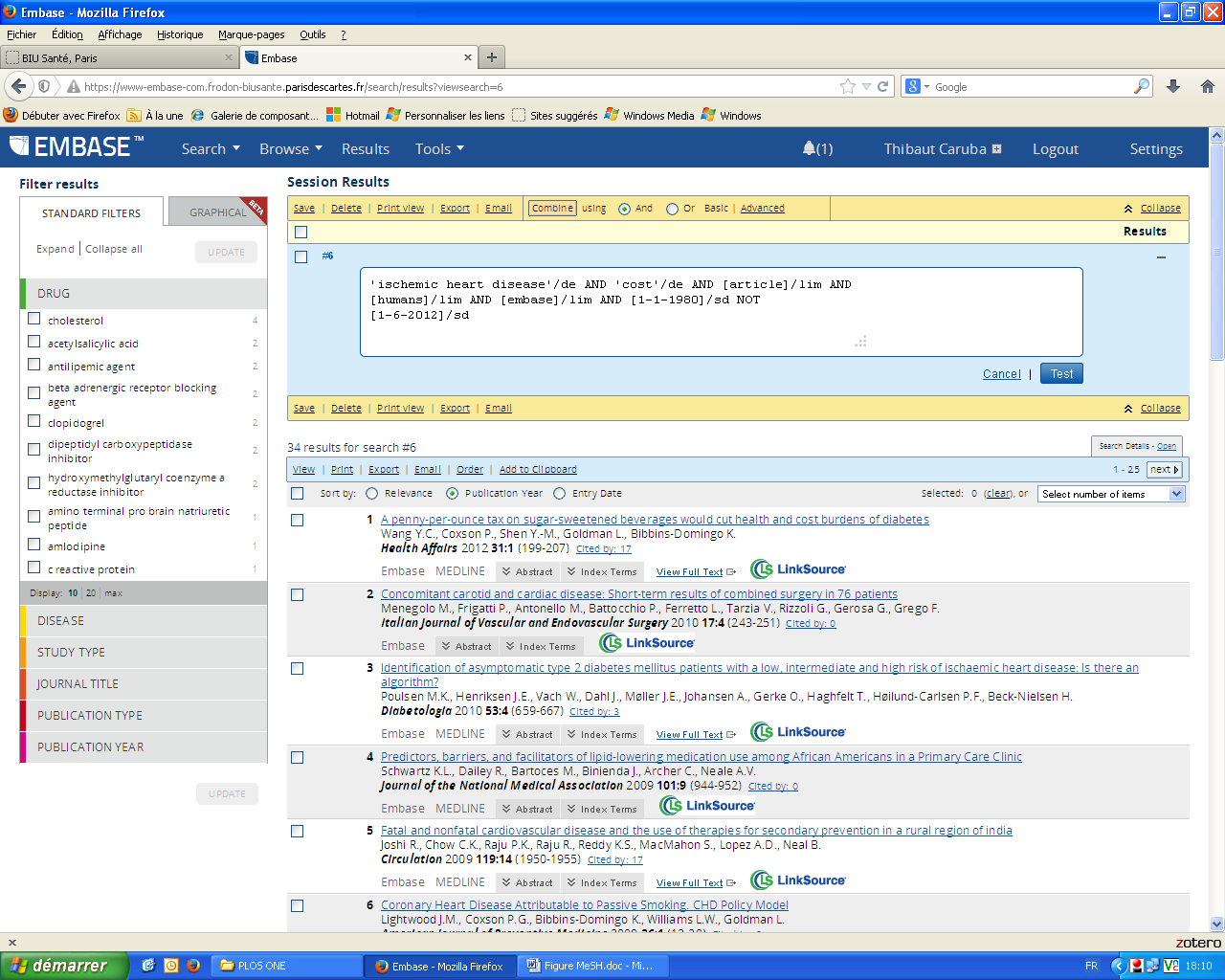


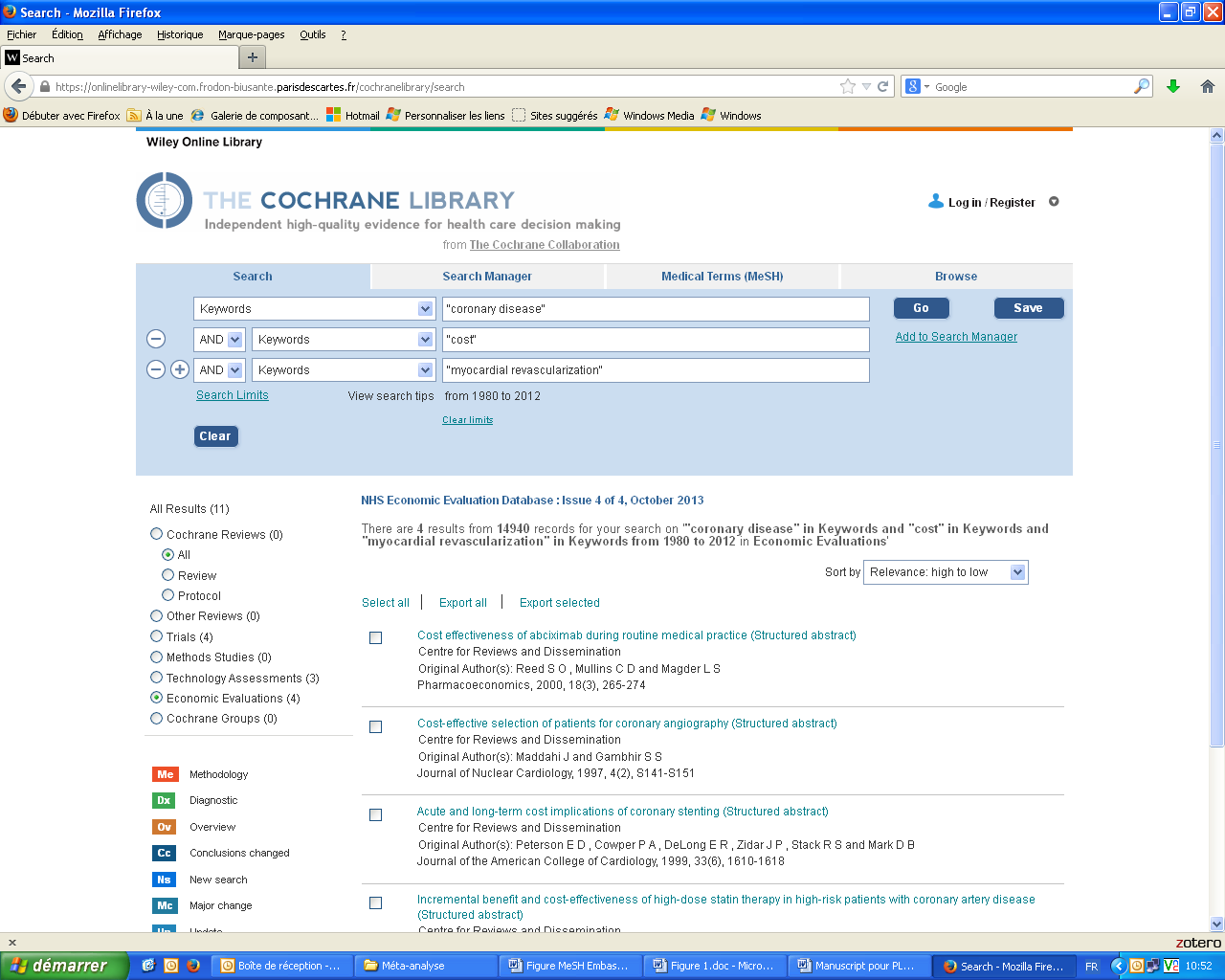

Supplement: Figure S1 — Search strategy in Medline, Embase and Cochrane library. (DOC) [file pone.0098371.s001.doc]
