# Supplementary material for: Treatment for Stable Coronary Artery Disease: A Network Meta-Analysis of Cost-Effectiveness Studies
Source: PLoS One. 2014 Jun 4;9(6):e98371. doi: 10.1371/journal.pone.0098371 (PMC4045726; doi:10.1371/journal.pone.0098371)
Supplement: Figure S2 — Checklist of clinical quality assessment according to the Heart Collaborative Review Group. (DOC) [file pone.0098371.s002.doc]

Figure S2: Checklist of clinical quality assessment according to the Heart Collaborative Review Group.

***Adequacy of the randomisation process***

A - Adequate sequence generation is reported (such as computer generated random numbers and random number tables, whilst inadequate approaches will include the use of alternation, case record numbers, birth dates or days of the week);

B - Did not specify one of the adequate reported methods in (A) but mentioned randomisation method;

C - Other methods of allocation that appear to be unbiased.

***Adequacy of the allocation concealment process***

A - Adequate measures to conceal allocations. Concealment will be deemed adequate where randomisation is centralised or pharmacy - controlled, or where the following are used: serially numbered containers, on - site computer - based systems where assignment is unreadable until after allocation, other methods with robust methods to prevent foreknowledge of the allocation sequence to clinicians and patients;

B - Unclearly concealed trials, in which the authors either did not report an allocation concealment approach at all, or reported an approach that did not fall into one of the categories in (A);

C - Inadequately concealed trials, in which method of allocation is not concealed. Inadequate approaches will include: the use of alternation, case record numbers, days of the week, open random number lists and serially numbered envelopes even if opaque.

***Potential for selection bias after allocation***

A - Studies where an intention to treat analysis is possible and few exclusions (with adequate reporting of these exclusions);

B - Studies which reported exclusions as reported in (A), but exclusions were less than 10 percent;

C - No reporting of exclusions; exclusions of 10 percent or more or wide differences in exclusions between groups.

***Adequacy of masking (see note on practicality of masking above)***

A - Double (or triple) blind;

B - Single blind;

C - Non - blind;

D - Unclear.
