# Supplementary material for: Treatment for Stable Coronary Artery Disease: A Network Meta-Analysis of Cost-Effectiveness Studies
Source: PLoS One. 2014 Jun 4;9(6):e98371. doi: 10.1371/journal.pone.0098371 (PMC4045726; doi:10.1371/journal.pone.0098371)
Supplement: Table S1 — Clinical quality assessment. (DOC) [file pone.0098371.s004.doc]

Table S1: Clinical quality assessment.

| **Etude** | **Adequacy of the randomisation process** | **Adequacy of the allocation concealment process** | **Potential for selection bias after allocation** | **Adequacy of masking (see note on practicality of masking above)** |
| --- | --- | --- | --- | --- |
| ACME | B | B | A | C |
| ARTS | A | A | A | C |
| BENESTENT II | A | A | A | C |
| COURAGE | A | A | A | C |
| EAST | B | B | A | C |
| ENDEAVOR II | A | B | A | A |
| ERACI | B | B | A | C |
| MASS II | A | A | A | C |
| RAVEL | A | A | A | A |
| RITA 2 | B | B | A | C |
| SIRIUS | A | A | A | A |
| SoS | A | A | A | C |
| STRESS | A | A | A | C |
| SYNTAX | A | A | A | C |
| TAXUS IV | A | A | A | A |
