# Supplementary material for: Treatment for Stable Coronary Artery Disease: A Network Meta-Analysis of Cost-Effectiveness Studies
Source: PLoS One. 2014 Jun 4;9(6):e98371. doi: 10.1371/journal.pone.0098371 (PMC4045726; doi:10.1371/journal.pone.0098371)
Supplement: Table S2 — Economical quality assessment. (DOC) [file pone.0098371.s005.doc]

Table S2: Economical quality assessment.

| **Etude** | **1** | **2** | **3** | **4** | **5** | **6** | **7** | **8** | **9** | **10** | **11** | **12** | **13** | **14** | **15** | **16** | **17** | **18** | **19** | **20** | **21** | **22** | **23** | **24** | **25** | **26** | **27** | **28** | **29** | **30** | **31** | **32** | **33** | **34** | **35** |
| --- | --- | --- | --- | --- | --- | --- | --- | --- | --- | --- | --- | --- | --- | --- | --- | --- | --- | --- | --- | --- | --- | --- | --- | --- | --- | --- | --- | --- | --- | --- | --- | --- | --- | --- | --- |
| **ACME** | O | O | O | O | O | O | O | O | O | NA | O | O | O | NA | NA | O | O | O | N | NA | NA | O | O | O | NA | N | O | O | O | O | N | N | O | O | O |
| **ARTS** | O | O | O | O | O | O | O | O | O | NA | O | O | O | NA | NA | O | O | N | N | NA | NA | O | N | N | N | N | N | N | N | O | O | O | O | O | O |
| **BENESTENT II** | O | O | O | O | O | O | O | O | O | NA | O | O | O | NA | NA | O | O | O | N | NA | NA | O | NA | NA | NA | O | N | N | N | O | O | O | O | O | O |
| **COURAGE** | O | O | O | O | O | O | O | O | O | NA | O | O | O | NA | NA | O | O | O | O | NA | NA | O | O | O | O | O | O | O | O | O | O | O | O | O | O |
| **EAST** | O | O | O | O | O | O | O | O | O | NA | O | O | O | NA | NA | O | O | N | N | NA | NA | O | N | N | N | N | N | N | N | O | O | O | O | O | O |
| **ENDEAVOR II** | O | O | O | O | O | O | O | O | O | NA | O | O | O | NA | NA | O | O | N | O | NA | NA | O | O | NC | N | O | N | N | N | O | O | O | O | O | O |
| **ERACI** | O | O | O | O | O | O | O | O | O | NA | O | O | O | NA | NA | O | N | O | N | NA | NA | O | N | NA | NA | N | N | N | N | O | O | O | O | O | O |
| **MASS II** | O | O | O | O | O | O | O | O | O | NA | O | O | O | NA | NA | O | O | O | N | NA | NA | O | NA | NA | NA | N | N | N | N | O | O | O | O | O | O |
| **RAVEL** | O | O | O | O | O | O | O | O | O | NA | O | O | O | NA | NA | O | O | O | N | NA | NA | O | NA | NA | NA | O | O | N | N | O | O | O | O | O | O |
| **RITA 2** | O | O | O | O | O | O | O | O | O | NA | O | O | O | NA | NA | O | O | O | N | NA | NA | O | O | N | N | O | O | O | O | O | O | O | O | O | O |
| **SIRIUS** | O | O | O | O | O | O | O | O | O | NA | O | O | O | NA | NA | O | O | O | N | NA | NA | O | NA | NA | NA | O | O | O | O | O | O | O | O | O | O |
| **SoS** | O | O | O | O | O | O | O | O | O | NA | O | O | O | NA | NA | O | O | O | N | NA | NA | O | NA | NA | NA | O | N | N | N | O | O | O | O | O | O |
| **STRESS** | O | O | O | O | O | O | O | O | O | NA | O | O | O | NA | NA | O | O | O | N | NA | NA | O | NA | NA | NA | O | N | N | N | O | O | O | O | O | O |
| **SYNTAX** | O | O | O | O | O | O | O | O | O | NA | O | O | O | NA | NA | O | O | O | N | NA | NA | O | NA | NA | NA | O | O | N | N | O | O | O | O | O | O |
| **TAXUS IV** | O | O | O | O | O | O | O | O | O | NA | O | O | O | NA | NA | O | O | N | O | NA | NA | O | NA | N | N | O | O | O | O | O | O | O | O | O | O |

Y: yes ; N : no ; NC : not clear ; NA : not appropriate.
