# Supplementary material for: Treatment for Stable Coronary Artery Disease: A Network Meta-Analysis of Cost-Effectiveness Studies
Source: PLoS One. 2014 Jun 4;9(6):e98371. doi: 10.1371/journal.pone.0098371 (PMC4045726; doi:10.1371/journal.pone.0098371)
Supplement: Table S3 — Sensitivity analyse for the comparison MT versus CABG versus PTCA versus BMS versus DES. (DOC) [file pone.0098371.s006.doc]

Table S3: Sensitivity analyse for the comparison MT *versus* CABG *versus* PTCA *versus* BMS *versus* DES.

|  |  | **MT** | **CABG** | **PTCA** | **BMS** | **DES** | **p-value**  **MT *versus* CABG *versus* PTCA *versus* BMS *versus* DES** |
| --- | --- | --- | --- | --- | --- | --- | --- |
| 1 year | Cost (SD) | 3 069 (0) | 27 098 (11 656) | - | 15 183 (3 117) | 25 739 (9 785) | <0.0001 |
|  | number of studies | 1 | 4 | 0 | 6 | 4 |  |
| 3 years | Cost (SD) | 13 864 (5 422) | 23 596 (0) | 10 739 (1 810) | 27 277 (4 508) | - | <0.0001 |
|  | number of studies | 3 | 1 | 2 | 2 | 0 |  |

MT: medical therapy, PTCA: percutaneous coronary angioplasty, CABG: coronary artery bypass graft, DES: drug eluting stent, BMS: bare metal stent, HR: hazard ratio, SD: standard deviation
