# Supplementary material for: Treatment for Stable Coronary Artery Disease: A Network Meta-Analysis of Cost-Effectiveness Studies
Source: PLoS One. 2014 Jun 4;9(6):e98371. doi: 10.1371/journal.pone.0098371 (PMC4045726; doi:10.1371/journal.pone.0098371)
Supplement: Table S4 — List of selected or rejected articles and reason in case of exclusion. (DOC) [file pone.0098371.s007.doc]

Table S4: List of selected or rejected articles and reason in case of exclusion

| **Title, Authors, Journal** | | **Article selected or excluded and main reason for exclusion** |
| --- | --- | --- |
| **MEDLINE (PubMed)** | | |
| 1 | Cost-effectiveness of everolimus- versus paclitaxel-eluting stents for patients undergoing percutaneous coronary revascularization (from the SPIRIT-IV Trial). Amin AP, Reynolds MR, Lei Y, Magnuson EA, Vilain K, Durtschi AJ, Simonton CA, Stone GW, Cohen DJ. Am J Cardiol. 2012 Sep 15;110(6):765-70. | Excluded : 2 treatments identical (2 drug eluting stents) |
| 2 | Rationale and design of the coronary artery bypass grafting surgery off or on pump revascularization study: a large international randomized trial in cardiac surgery. Lamy A, Devereaux PJ, Prabhakaran D, Hu S, Piegas LS, Straka Z, Paolasso E, Taggart D, Lanas F, Akar AR, Jain A, Noiseux N, Ou Y, Chrolavicius S, Ng J, Yusuf S. Am Heart J. 2012 Jan;163(1):1-6. | Excluded : unconventional treatment |
| 3 | Randomized comparison of cost-saving and effectiveness of oral rapamycin plus bare-metal stents with drug-eluting stents: three-year outcome from the randomized oral rapamycin in Argentina (ORAR) III trial. Rodriguez AE, Rodriguez-Granillo AM, Antoniucci D, Mieres J, Fernandez-Pereira C, Rodriguez-Granillo GA, Santaera O, Rubilar B, Palacios IF, Serruys PW; ORAR III Investigators. Catheter Cardiovasc Interv. 2012 Sep 1;80(3):385-94 | Excluded : unconventional treatment |
| 4 | Endoscopic Saphenous harvesting with an Open CO2 System (ESOS) trial for coronary artery bypass grafting surgery: study protocol for a randomized controlled trial. Campanella A, Bergamasco L, Macri L, Asioli S, Devotini R, Scipioni S, Barbaro S, Rispoli P, Rinaldi M. Trials. 2011 Nov 18;12:243. | Excluded : other intervention was studied |
| 5 | Estimation of lung vital capacity before and after coronary artery bypass grafting surgery: a comparison of incentive spirometer and ventilometry. Pinheiro AC, Novais MC, Neto MG, Rodrigues MV, de Souza Rodrigues E Jr, Aras R Jr, Carvalho VO. J Cardiothorac Surg. 2011 May 12;6:70 | Excluded : other intervention was studied |
| 6 | Economic outcomes of percutaneous coronary intervention with drug-eluting stents versus bypass surgery for patients with left main or three-vessel coronary artery disease: one-year results from the SYNTAX trial. Cohen DJ, Lavelle TA, Van Hout B, Li H, Lei Y, Robertus K, Pinto D, Magnuson EA, Mcgarry TF, Lucas SK, Horwitz PA, Henry CA, Serruys PW, Mohr FW, Kappetein AP. Catheter Cardiovasc Interv. 2012 Feb 1;79(2):198-209 | selected |
| 7 | The impact of race/ethnicity on baseline characteristics and the burden of coronary atherosclerosis in the Bypass Angioplasty Revascularization Investigation 2 Diabetes trial. Beohar N, Davidson CJ, Massaro EM, Srinivas VS, Sansing VV, Zonszein J, Davis AM, Helmy T, Lopes NH, Thomas SB, Brooks MM. Am Heart J. 2011 Apr;161(4):755-63 | Excluded : population with different inclusion criteria (diabetes mellitus) |
| 8 | Effects of optimal medical treatment with or without coronary revascularization on angina and subsequent revascularizations in patients with type 2 diabetes mellitus and stable ischemic heart disease. Dagenais GR, Lu J, Faxon DP, Kent K, Lago RM, Lezama C, Hueb W, Weiss M, Slater J, Frye RL; Bypass Angioplasty Revascularization Investigation 2 Diabetes (BARI 2D) Study Group. Circulation. 2011 Apr 12;123(14):1492-500. | Excluded : population with different inclusion criteria (diabetes mellitus) |
| 9 | Long-term impact of secondary preventive treatments in patients with stable angina. Vokó Z, de Brouwer S, Lubsen J, Danchin N, Otterstad JE, Dunselman PH, Kirwan BA. Eur J Epidemiol. 2011 May;26(5):375-83. | Excluded : incomplete data (no economic data available at 1 and/or 3 years) |
| 10 | The cost-effectiveness of percutaneous coronary intervention as a function of angina severity in patients with stable angina. Zhang Z, Kolm P, Boden WE, Hartigan PM, Maron DJ, Spertus JA, O'Rourke RA, Shaw LJ, Sedlis SP, Mancini GB, Berman DS, Dada M, Teo KK, Weintraub WS. Circ Cardiovasc Qual Outcomes. 2011 Mar;4(2):172-82. | Excluded : doubloon (data used in another reference) |
| 11 | Economic evaluation of fractional flow reserve-guided percutaneous coronary intervention in patients with multivessel disease. Fearon WF, Bornschein B, Tonino PA, Gothe RM, Bruyne BD, Pijls NH, Siebert U; Fractional Flow Reserve Versus Angiography for Multivessel Evaluation (FAME) Study Investigators. Circulation. 2010 Dec 14;122(24):2545-50. | Excluded : other intervention was studied |
| 12 | The generalizability of participants in Veterans Affairs Cooperative Studies Program 474, a multi-site randomized cardiac bypass surgery trial. Wagner TH, Holman W, Lee K, Sethi G, Ananth L, Thai H, Goldman S. Contemp Clin Trials. 2011 Mar;32(2):260-6. | Excluded : 2 treatments identical (coronary artery bypass surgery) |
| 13 | Percutaneous coronary intervention with oral sirolimus and bare metal stents has comparable safety and efficacy to treatment with drug eluting stents, but with significant cost saving: long-term follow-up results from the randomised, controlled ORAR III (Oral Rapamycin in ARgentina) study. Rodriguez AE, Maree A, Tarragona S, Fernandez-Pereira C, Santaera O, Rodriguez Granillo AM, Rodriguez-Granillo GA, Russo-Felssen M, Kukreja N, Antoniucci D, Palacios IF, Serruys PW; ORAR III Investigators. EuroIntervention. 2009 Jun;5(2):255-64. | Excluded : follow up doesn't correspond to (less than 1 year or different than 1 and/or 3 years) |
| 14 | Intensive multifactorial intervention for stable coronary artery disease: optimal medical therapy in the COURAGE (Clinical Outcomes Utilizing Revascularization and Aggressive Drug Evaluation) trial. Maron DJ, Boden WE, O'Rourke RA, Hartigan PM, Calfas KJ, Mancini GB, Spertus JA, Dada M, Kostuk WJ, Knudtson M, Harris CL, Sedlis SP, Zoble RG, Title LM, Gosselin G, Nawaz S, Gau GT, Blaustein AS, Bates ER, Shaw LJ, Berman DS, Chaitman BR, Weintraub WS, Teo KK; COURAGE Trial Research Group. J Am Coll Cardiol. 2010 Mar 30;55(13):1348-58. | Excluded : follow up doesn't correspond to (less than 1 year or different than 1 and/or 3 years) |
| 15 | Multicentre randomised controlled trial of the clinical and cost-effectiveness of a bypass-surgery-first versus a balloon-angioplasty-first revascularisation strategy for severe limb ischaemia due to infrainguinal disease. The Bypass versus Angioplasty in Severe Ischaemia of the Leg (BASIL) trial. Bradbury AW, Adam DJ, Bell J, Forbes JF, Fowkes FG, Gillespie I, Raab G, Ruckley CV. Health Technol Assess. 2010 Mar;14(14):1-210, iii-iv | Excluded : other intervention was studied |
| 16 | Late safety, efficacy, and cost-effectiveness of a zotarolimus-eluting stent compared with a paclitaxel-eluting stent in patients with de novo coronary lesions: 2-year follow-up from the ENDEAVOR IV trial (Randomized, Controlled Trial of the Medtronic Endeavor Drug [ABT-578] Eluting Coronary Stent System Versus the Taxus Paclitaxel-Eluting Coronary Stent System in De Novo Native Coronary Artery Lesions). Leon MB, Kandzari DE, Eisenstein EL, Anstrom KJ, Mauri L, Cutlip DE, Nikolsky E, O'Shaughnessy C, Overlie PA, Kirtane AJ, McLaurin BT, Solomon SL, Douglas JS Jr, Popma JJ; ENDEAVOR IV Investigators. JACC Cardiovasc Interv. 2009 Dec;2(12):1208-18. | Excluded : 2 treatments identical (2 drug eluting stents) |
| 17 | Long-term clinical and economic analysis of the Endeavor zotarolimus-eluting stent versus the cypher sirolimus-eluting stent: 3-year results from the ENDEAVOR III trial (Randomized Controlled Trial of the Medtronic Endeavor Drug [ABT-578] Eluting Coronary Stent System Versus the Cypher Sirolimus-Eluting Coronary Stent System in De Novo Native Coronary Artery Lesions). Eisenstein EL, Leon MB, Kandzari DE, Mauri L, Edwards R, Kong DF, Cowper PA, Anstrom KJ; ENDEAVOR III Investigators. JACC Cardiovasc Interv. 2009 Dec;2(12):1199-207. | Excluded : 2 treatments identical (2 drug eluting stents) |
| 18 | Long-term clinical and economic analysis of the Endeavor drug-eluting stent versus the Driver bare-metal stent: 4-year results from the ENDEAVOR II trial (Randomized Controlled Trial to Evaluate the Safety and Efficacy of the Medtronic AVE ABT-578 Eluting Driver Coronary Stent in De Novo Native Coronary Artery Lesions). Eisenstein EL, Wijns W, Fajadet J, Mauri L, Edwards R, Cowper PA, Kong DF, Anstrom KJ. JACC Cardiovasc Interv. 2009 Dec;2(12):1178-87 | selected |
| 19 | Hyperbaric oxygen preconditioning improves myocardial function, reduces length of intensive care stay, and limits complications post coronary artery bypass graft surgery. Yogaratnam JZ, Laden G, Guvendik L, Cowen M, Cale A, Griffin S. Cardiovasc Revasc Med. 2010 Jan-Mar;11(1):8-19. | Excluded : other intervention was studied |
| 20 | A clinical and angiographic study of the XIENCE V everolimus-eluting coronary stent system in the treatment of patients with multivessel coronary artery disease. Study design and rationale of the EXECUTIVE trial. Ribichini F, Ansalone G, Bartorelli A, Beqaraj F, Berni A, Colangelo S, D'Amico M, Della Rovere F, Fiscella A, Gabrielli G, Indolfi C, La Vecchia L, Loschiavo P, Marinoni G, Marzocchi A, Milazzo D, Romano M, Sangiorgio P, Sheiban I, Tamburino C, Tuccillo B, Villani R, Cappi B, Quijada MJ, Vassanelli C; EXECUTIVE Trial Investigators. J Cardiovasc Med (Hagerstown). 2010 Apr;11(4):299-309. | Excluded : it was a protocol |
| 21 | Screening asymptomatic patients with diabetes for unknown coronary artery disease: does it reduce risk? An open-label randomized trial comparing a strategy based on exercise testing aimed at revascularization with management based on pharmacological/behavioural treatment of traditional risk factors. DADDY-D Trial (Does coronary Atherosclerosis Deserve to be Diagnosed and treated early in Diabetics?). Turrini F, Messora R, Giovanardi P, Tondi S, Magnavacchi P, Cavani R, Tosoni G, Cappelli C, Pellegrini E, Romano S, Baldini A, Zennaro RG, Bondi M. Trials. 2009 Dec 23;10:119. | Excluded : population with different inclusion criteria (diabetes mellitus) |
| 22 | Influence of an early recovery telehealth intervention on physical activity and functioning after coronary artery bypass surgery among older adults with high disease burden. Barnason S, Zimmerman L, Schulz P, Tu C. Heart Lung. 2009 Nov-Dec;38(6):459-68. | Excluded : 2 treatments identical (coronary artery bypass surgery) |
| 23 | Patient satisfaction is comparable to early discharge versus overnight observation after elective percutaneous coronary intervention. Glaser R, Gertz Z, Matthai WH, Wilensky RL, Weiner M, Kolansky D, Hirshfeld J Jr, Herrmann H. J Invasive Cardiol. 2009 Sep;21(9):464-7. | Excluded : other intervention was studied (eraly discharge versus overnight observation after PCI) |
| 24 | The impact of avoiding cardiopulmonary by-pass during coronary artery bypass surgery in elderly patients: the Danish On-pump Off-pump Randomisation Study (DOORS). Houlind K, Kjeldsen BJ, Madsen SN, Rasmussen BS, Holme SJ, Schmidt TA, Haahr PE, Mortensen PE; DOORS study group. Trials. 2009 Jul 4;10:47 | Excluded : unconventional treatment |
| 25 | Clinical insights from the Treating to New Targets trial. Waters DD. Prog Cardiovasc Dis. 2009 May-Jun;51(6):487-502 | Excluded : other intervention was studied |
| 26 | A multicenter, randomized study to test immunosuppressive therapy with oral prednisone for the prevention of restenosis after percutaneous coronary interventions: cortisone plus BMS or DES versus BMS alone to eliminate restenosis (CEREA-DES) - study design and rationale. Ribichini F, Tomai F, De Luca G, Boccuzzi G, Presbitero P, Pesarini G, Ferrero V, Ghini AS, Pastori F, De Luca L, Zavalloni D, Soregaroli D, Garbo R, Franchi E, Marino P, Minelli M, Vassanelli C. J Cardiovasc Med (Hagerstown). 2009 Feb;10(2):192-9. | Excluded : it was a protocol |
| 27 | Quality of life after late invasive therapy for occluded arteries. Mark DB, Pan W, Clapp-Channing NE, Anstrom KJ, Ross JR, Fox RS, Devlin GP, Martin CE, Adlbrecht C, Cowper PA, Ray LD, Cohen EA, Lamas GA, Hochman JS; Occluded Artery Trial Investigators. N Engl J Med. 2009 Feb 19;360(8):774-83. | Excluded : population with different inclusion criteria (persistent occluded arterial) |
| 28 | The Bypassing the Blues treatment protocol: stepped collaborative care for treating post-CABG depression. Rollman BL, Belnap BH, LeMenager MS, Mazumdar S, Schulberg HC, Reynolds CF 3rd. Psychosom Med. 2009 Feb;71(2):217-30. | Excluded : other intervention was studied |
| 29 | Fractional flow reserve versus angiography for guiding percutaneous coronary intervention. Tonino PA, De Bruyne B, Pijls NH, Siebert U, Ikeno F, van' t Veer M, Klauss V, Manoharan G, Engstrøm T, Oldroyd KG, Ver Lee PN, MacCarthy PA, Fearon WF; FAME Study Investigators. N Engl J Med. 2009 Jan 15;360(3):213-24 | Excluded : other intervention was studied |
| 30 | Comparative costs between myocardial revascularization with or without extracorporeal circulation. Girardi PB, Hueb W, Nogueira CR, Takiuti ME, Nakano T, Garzillo CL, Paulitsch Fda S, Góis AF, Lopes NH, Stolf NA. Arq Bras Cardiol. 2008 Dec;91(6):340-6. | Excluded : unconventional treatment |
| 31 | Industry-sponsored negative trials and the potential pitfalls of post hoc analysis. Tribble CG. Arch Surg. 2008 Oct;143(10):933-4. | Excluded : other intervention was studied |
| 32 | Long-term economic outcomes associated with intensive versus moderate lipid-lowering therapy in coronary artery disease: results from the Treating to New Targets (TNT) Trial. Mark DB, Knight JD, Cowper PA, Davidson-Ray L, Anstrom KJ. Am Heart J. 2008 Oct;156(4):698-705. | Excluded : other intervention was studied |
| 33 | Cost-effectiveness of percutaneous coronary intervention in optimally treated stable coronary patients. Weintraub WS, Boden WE, Zhang Z, Kolm P, Zhang Z, Spertus JA, Hartigan P, Veledar E, Jurkovitz C, Bowen J, Maron DJ, O'Rourke R, Dada M, Teo KK, Goeree R, Barnett PG; Department of Veterans Affairs Cooperative Studies Program No. 424 (COURAGE Trial) Investigators and Study Coordinators. Circ Cardiovasc Qual Outcomes. 2008 Sep;1(1):12-20. | selected |
| 34 | Acute normovolaemic haemodilution with crystalloids in coronary artery bypass graft surgery: a preliminary survey of haemostatic markers. Jalali A, Naseri MH, Chalian M, Dolatabadi HL. Acta Cardiol. 2008 Jun;63(3):335-9 | Excluded : other intervention was studied |
| 35 | Rationale and design of the Post-MI FREEE trial: a randomized evaluation of first-dollar drug coverage for post-myocardial infarction secondary preventive therapies. Choudhry NK, Brennan T, Toscano M, Spettell C, Glynn RJ, Rubino M, Schneeweiss S, Brookhart AM, Fernandes J, Mathew S, Christiansen B, Antman EM, Avorn J, Shrank WH. Am Heart J. 2008 Jul;156(1):31-6 | Excluded : it was a protocol |
| 36 | Value and limitations of target-vessel ischemia in predicting late clinical events after drug-eluting stent implantation. Zellweger MJ, Kaiser C, Brunner-La Rocca HP, Buser PT, Osswald S, Weiss P, Mueller-Brand J, Pfisterer ME; BASKET Investigators. J Nucl Med. 2008 Apr;49(4):550-6. | Excluded : population with different inclusion criteria (21% havec a STMI) |
| 37 | A randomised controlled trial to evaluate a nurse-led programme of support and lifestyle management for patients awaiting cardiac surgery 'Fit for surgery: Fit for life' study. Goodman H, Parsons A, Davison J, Preedy M, Peters E, Shuldham C, Pepper J, Cowie MR. Eur J Cardiovasc Nurs. 2008 Sep;7(3):189-95 | Excluded : other intervention was studied |
| 38 | Amiodarone cost effectiveness in preventing atrial fibrillation after coronary artery bypass graft surgery. Zebis LR, Christensen TD, Kristiansen IS, Hjortdal VE. Ann Thorac Surg. 2008 Jan;85(1):28-32. | Excluded : other intervention was studied |
| 39 | Cost-effectiveness of sirolimus-eluting stents compared with vascular brachytherapy for the treatment of in-stent restenosis. Reynolds MR, Pinto DS, Shi C, Walczak J, Berezin R, Holmes DR Jr, Cohen DJ. Am Heart J. 2007 Dec;154(6):1221-7. | Excluded : other intervention was studied |
| 40 | The rationale and design of the Surgical Treatment for Ischemic Heart Failure (STICH) trial. Velazquez EJ, Lee KL, O'Connor CM, Oh JK, Bonow RO, Pohost GM, Feldman AM, Mark DB, Panza JA, Sopko G, Rouleau JL, Jones RH; STICH Investigators. J Thorac Cardiovasc Surg. 2007 Dec;134(6):1540-7. | Excluded : it was a protocol |
| 41 | Cost-effectiveness of functional cardiac testing in the diagnosis and management of coronary artery disease: a randomised controlled trial. The CECaT trial. Sharples L, Hughes V, Crean A, Dyer M, Buxton M, Goldsmith K, Stone D. Health Technol Assess. 2007 Dec;11(49):iii-iv, ix-115. | Excluded : other intervention was studied |
| 42 | Cost-effectiveness of clopidogrel in acute coronary syndromes in Canada: a long-term analysis based on the CURE trial. Kolm P, Yuan Y, Veledar E, Mehta SR, O'Brien JA, Weintraub WS. Can J Cardiol. 2007 Nov;23(13):1037-42. | Excluded : population with different inclusion criteria (acute coronary syndrom) |
| 43 | Cost-effectiveness of drug-eluting stents in patients at high or low risk of major cardiac events in the Basel Stent KostenEffektivitäts Trial (BASKET): an 18-month analysis. Brunner-La Rocca HP, Kaiser C, Bernheim A, Zellweger MJ, Jeger R, Buser PT, Osswald S, Pfisterer M; BASKET Investigators. Lancet. 2007 Nov 3;370(9598):1552-9. | Excluded : population with different inclusion criteria (21% havec a STEMI) |
| 44 | Rationale and design of the Fractional Flow Reserve versus Angiography for Multivessel Evaluation (FAME) study. Fearon WF, Tonino PA, De Bruyne B, Siebert U, Pijls NH; FAME Study Investigators. Am Heart J. 2007 Oct;154(4):632-6. | Excluded : it was a protocol |
| 45 | Randomized trial comparing same-day discharge with overnight hospital stay after percutaneous coronary intervention: results of the Elective PCI in Outpatient Study (EPOS). Heyde GS, Koch KT, de Winter RJ, Dijkgraaf MG, Klees MI, Dijksman LM, Piek JJ, Tijssen JG. Circulation. 2007 May 1;115(17):2299-306. | Excluded : follow up doesn't correspond to (less than 1 year or different than 1 and/or 3 years) |
| 46 | Targeted stent use in clinical practice based on evidence from the Basel Stent Cost Effectiveness Trial (BASKET). Brunner-La Rocca HP, Kaiser C, Pfisterer M; BASKET Investigators. Eur Heart J. 2007 Mar;28(6):719-25. | Excluded : population with different inclusion criteria (21% havec a STEMI) |
| 47 | Stress testing and troponin in unstable coronary syndromes: the status trial-clinical outcomes and resource use. Estrada JN, Rolandi F, Bansilal S, Averbuj P, Natale E, Zafar MU, Santra M, Barbiere J, Chesebro JH, Farkouh ME. Am Heart Hosp J. 2006 Fall;4(4):252-8 | Excluded : other intervention was studied |
| 48 | Cost-effectiveness of coronary artery bypass grafts versus percutaneous coronary intervention for revascularization of high-risk patients. Stroupe KT, Morrison DA, Hlatky MA, Barnett PG, Cao L, Lyttle C, Hynes DM, Henderson WG; Investigators of Veterans Affairs Cooperative Studies Program #385 (AWESOME: Angina With Extremely Serious Operative Mortality Evaluation). Circulation. 2006 Sep 19;114(12):1251-7. | Excluded : population with different inclusion criteria (patients with myocardial infarction in the previous 7 days) |
| 49 | Cost-effectiveness of nurse practitioner management of hypercholesterolemia following coronary revascularization. Paez KA, Allen JK. J Am Acad Nurse Pract. 2006 Sep;18(9):436-44. | Excluded : other intervention was studied |
| 50 | Effect of low-dose amiodarone and magnesium combination on atrial fibrillation after coronary artery surgery. Cagli K, Ozeke O, Ergun K, Budak B, Demirtas E, Birincioglu CL, Pac M. J Card Surg. 2006 Sep-Oct;21(5):458-64. | Excluded : other intervention was studied |
| 51 | Are drug-eluting stents cost-effective when compared with bare-metal stents in a real-world setting? Moussa I. Nat Clin Pract Cardiovasc Med. 2006 Apr;3(4):192-3. | Excluded : no randomization |
| 52 | Is it necessary to use a drain after harvesting radial artery? A randomized prospective study. Oz BS, Bolcal C, Kucukarslan N, Kuralay E, Yildirim V, Tatar H. J Card Surg. 2006 Mar-Apr;21(2):155-7. | Excluded : other intervention was studied |
| 53 | Effectiveness of "direct" stenting without balloon predilatation (from the Multilink Tetra Randomised European Direct Stent Study [TRENDS]). Dawkins KD, Chevalier B, Suttorp MJ, Thuesen L, Benit E, Bethencourt A, Morjaria U, Veldhof S, Dorange C, van Weert A; TRENDS Investigators. Am J Cardiol. 2006 Feb 1;97(3):316-21. | Excluded : follow up doesn't correspond to (less than 1 year or different than 1 and/or 3 years) |
| 54 | Short-stay intensive care after coronary artery bypass surgery: randomized clinical trial on safety and cost-effectiveness. van Mastrigt GA, Heijmans J, Severens JL, Fransen EJ, Roekaerts P, Voss G, Maessen JG. Crit Care Med. 2006 Jan;34(1):65-75. | Excluded : follow up doesn't correspond to (less than 1 year or different than 1 and/or 3 years) |
| 55 | Femoral access management: comparison between two different vascular closure devices after percutaneous coronary intervention. Legrand V, Doneux P, Martinez C, Gach O, Bellekens M. Acta Cardiol. 2005 Oct;60(5):482-8. | Excluded : other intervention was studied |
| 56 | Incremental cost-effectiveness of drug-eluting stents compared with a third-generation bare-metal stent in a real-world setting: randomised Basel Stent Kosten Effektivitäts Trial (BASKET). Kaiser C, Brunner-La Rocca HP, Buser PT, Bonetti PO, Osswald S, Linka A, Bernheim A, Zutter A, Zellweger M, Grize L, Pfisterer ME; BASKET Investigators. Lancet. 2005 Sep 10-16;366(9489):921-9. | Excluded : follow up doesn't correspond to (less than 1 year or different than 1 and/or 3 years) |
| 57 | Single vs multivessel treatment during primary angioplasty: results of the multicentre randomised HEpacoat for cuLPrit or multivessel stenting for Acute Myocardial Infarction (HELP AMI) Study. Di Mario C, Mara S, Flavio A, Imad S, Antonio M, Anna P, Emanuela P, Stefano DS, Angelo R, Stefania C, Anna F, Carmelo C, Antonio C, Monzini N, Bonardi MA. Int J Cardiovasc Intervent. 2004;6(3-4):128-33. | Excluded : population with different inclusion criteria (patients with acute myocardial infarction) |
| 58 | The impact of acute coronary syndrome on clinical, economic, and cardiac-specific health status after coronary artery bypass surgery versus stent-assisted percutaneous coronary intervention: 1-year results from the stent or surgery (SoS) trial. Zhang Z, Spertus JA, Mahoney EM, Booth J, Nugara F, Stables RH, Weintraub WS. Am Heart J. 2005 Jul;150(1):175-81. | Excluded : doubloon (data used in another reference) |
| 59 | Clinical and angiographic procedural and mid-term outcome with new versus reused balloon catheters in percutaneous coronary interventions. Unverdorben M, Degenhardt R, Erny D, Scholz M, Wagner E, Köhler H, Berthold HK, Vallbracht C. Indian Heart J. 2005 Mar-Apr;57(2):114-20. | Excluded : follow up doesn't correspond to (less than 1 year or different than 1 and/or 3 years) |
| 60 | Safety, efficacy, and cost of intraoperative cell salvage and autotransfusion after off-pump coronary artery bypass surgery: a randomized trial. Murphy GJ, Rogers CS, Lansdowne WB, Channon I, Alwair H, Cohen A, Caputo M, Angelini GD. J Thorac Cardiovasc Surg. 2005 Jul;130(1):20-8. | Excluded : other intervention was studied |
| 61 | The effect of oral prednisolone with chronic obstructive pulmonary disease undergoing coronary artery bypass surgery. Bingol H, Cingoz F, Balkan A, Kilic S, Bolcal C, Demirkilic U, Tatar H. J Card Surg. 2005 May-Jun;20(3):252-6. | Excluded : other intervention was studied |
| 62 | Outpatient coronary angioplasty: feasible and safe. Slagboom T, Kiemeneij F, Laarman GJ, van der Wieken R. Catheter Cardiovasc Interv. 2005 Apr;64(4):421-7. | Excluded : follow up doesn't correspond to (less than 1 year or different than 1 and/or 3 years) |
| 63 | Updated management of non-st-segment elevation acute coronary syndromes: selection of patients for low-cost care: an analysis of outcome and cost effectiveness. Conti A, Pieralli F, Sammicheli L, Camaiti A, Vanni S, Grifoni S, Dovellini E, Antoniucci D, Squillantini G, Mazzuoli F, Colombo G. Med Sci Monit. 2005 Mar;11(3):CR100-8. | Excluded : population with different inclusion criteria (acute coronary syndrom) |
| 64 | A short course of cardiac rehabilitation program is highly cost effective in improving long-term quality of life in patients with recent myocardial infarction or percutaneous coronary intervention. Yu CM, Lau CP, Chau J, McGhee S, Kong SL, Cheung BM, Li LS. Arch Phys Med Rehabil. 2004 Dec;85(12):1915-22. | Excluded : other intervention was studied |
| 65 | Cost-effectiveness of invasive versus medical management of elderly patients with chronic symptomatic coronary artery disease. Findings of the randomized trial of invasive versus medical therapy in elderly patients with chronic angina (TIME). Claude J, Schindler C, Kuster GM, Schwenkglenks M, Szucs T, Buser P, Osswald S, Kaiser C, Grädel C, Estlinbaum W, Rickenbacher P, Pfisterer M; Trial of Invasive versus Medical therapy in the Elderly (TIME) Investigators. Eur Heart J. 2004 Dec;25(24):2195-203. | Excluded : incomplete data (one of the study treatment was "revascularization" and did not distinguish angioplasty to surgery) |
| 66 | DECOPI (DEsobstruction COronaire en Post-Infarctus): a randomized multi-centre trial of occluded artery angioplasty after acute myocardial infarction. Steg PG, Thuaire C, Himbert D, Carrié D, Champagne S, Coisne D, Khalifé K, Cazaux P, Logeart D, Slama M, Spaulding C, Cohen A, Tirouvanziam A, Montély JM, Rodriguez RM, Garbarz E, Wijns W, Durand-Zaleski I, Porcher R, Brucker L, Chevret S, Chastang C; DECOPI Investigators. Eur Heart J. 2004 Dec;25(24):2187-94. | Excluded : population with different inclusion criteria (patients with persistent occluded arterial) |
| 67 | Cost-effectiveness of distal embolic protection for patients undergoing percutaneous intervention of saphenous vein bypass grafts: results from the SAFER trial. Cohen DJ, Murphy SA, Baim DS, Lavelle TA, Berezin RH, Cutlip DE, Ho KK, Kuntz RE; SAFER Trial Investigators. J Am Coll Cardiol. 2004 Nov 2;44(9):1801-8. | Excluded : other intervention was studied |
| 68 | Economic evaluation of bivalirudin with provisional glycoprotein IIB/IIIA inhibition versus heparin with routine glycoprotein IIB/IIIA inhibition for percutaneous coronary intervention: results from the REPLACE-2 trial. Cohen DJ, Lincoff AM, Lavelle TA, Chen HL, Bakhai A, Berezin RH, Jackman D, Sarembock IJ, Topol EJ. J Am Coll Cardiol. 2004 Nov 2;44(9):1792-800. | Excluded : other intervention was studied |
| 69 | Medical costs and quality of life 10 to 12 years after randomization to angioplasty or bypass surgery for multivessel coronary artery disease. Hlatky MA, Boothroyd DB, Melsop KA, Brooks MM, Mark DB, Pitt B, Reeder GS, Rogers WJ, Ryan TJ, Whitlow PL, Wiens RD. Circulation. 2004 Oct 5;110(14):1960-6. | Excluded : follow up doesn't correspond to (less than 1 year or different than 1 and/or 3 years) |
| 70 | Complete versus culprit vessel percutaneous coronary intervention in multivessel disease: a randomized comparison. Ijsselmuiden AJ, Ezechiels J, Westendorp IC, Tijssen JG, Kiemeneij F, Slagboom T, van der Wieken R, Tangelder G, Serruys PW, Laarman G. Am Heart J. 2004 Sep;148(3):467-74. | Excluded : one treatment studied was not conventionnal |
| 71 | Giving IV and oral amiodarone perioperatively for the prevention of postoperative atrial fibrillation in patients undergoing coronary artery bypass surgery: the GAP study. Kerstein J, Soodan A, Qamar M, Majid M, Lichstein E, Hollander G, Shani J. Chest. 2004 Sep;126(3):716-24. | Excluded : other intervention was studied |
| 72 | Willingness to pay for avoiding coronary restenosis and repeat revascularization: results from a contingent valuation study. Greenberg D, Bakhai A, Neumann PJ, Cohen DJ. Health Policy. 2004 Nov;70(2):207-16. | Excluded : economic model |
| 73 | A risk score to estimate the likelihood of coronary artery bypass surgery during the index hospitalization among patients with unstable angina and non-ST-segment elevation myocardial infarction. Sadanandan S, Cannon CP, Gibson CM, Murphy SA, DiBattiste PM, Braunwald E; TIMI Study Group. J Am Coll Cardiol. 2004 Aug 18;44(4):799-803. | Excluded : other intervention was studied |
| 74 | Cost-effectiveness of sirolimus-eluting stents for treatment of complex coronary stenoses: results from the Sirolimus-Eluting Balloon Expandable Stent in the Treatment of Patients With De Novo Native Coronary Artery Lesions (SIRIUS) trial. Cohen DJ, Bakhai A, Shi C, Githiora L, Lavelle T, Berezin RH, Leon MB, Moses JW, Carrozza JP Jr, Zidar JP, Kuntz RE; SIRIUS Investigators. Circulation. 2004 Aug 3;110(5):508-14. | selected |
| 75 | An economic evaluation of fluvastatin used for the prevention of cardiac events following successful first percutaneous coronary intervention in the UK. Scuffham PA, Chaplin S. Pharmacoeconomics. 2004;22(8):525-35. | Excluded : other intervention was studied |
| 76 | One year comparison of costs of coronary surgery versus percutaneous coronary intervention in the stent or surgery trial. Weintraub WS, Mahoney EM, Zhang Z, Chu H, Hutton J, Buxton M, Booth J, Nugara F, Stables RH, Dooley P, Collinson J, Stuteville M, Delahunty N, Wright A, Flather MD, De Cock E. Heart. 2004 Jul;90(7):782-8. | selected |
| 77 | Randomized comparison of rapid ambulation using radial, 4 French femoral access, or femoral access with AngioSeal closure. Reddy BK, Brewster PS, Walsh T, Burket MW, Thomas WJ, Cooper CJ. Catheter Cardiovasc Interv. 2004 Jun;62(2):143-9. | Excluded : other intervention was studied |
| 78 | Methods for the economic and quality of life supplement to the cilostazol for RESTenosis (CREST) trial. Weintraub WS, Foster J, Culler SD, Becker ER, Parker K, Zhang Z, Kolm P, Douglas JS Jr; Cilostazol for RESTenosis trial. J Invasive Cardiol. 2004 May;16(5):257-9. | Excluded : other intervention was studied |
| 79 | A multi-centre randomised controlled trial of minimally invasive direct coronary bypass grafting versus percutaneous transluminal coronary angioplasty with stenting for proximal stenosis of the left anterior descending coronary artery. Reeves BC, Angelini GD, Bryan AJ, Taylor FC, Cripps T, Spyt TJ, Samani NJ, Roberts JA, Jacklin P, Seehra HK, Culliford LA, Keenan DJ, Rowlands DJ, Clarke B, Stanbridge R, Foale R. Health Technol Assess. 2004 Apr;8(16):1-43. | Excluded : one treatment studied was not conventionnal |
| 80 | Percutaneous coronary angioplasty compared with exercise training in patients with stable coronary artery disease: a randomized trial. Hambrecht R, Walther C, Möbius-Winkler S, Gielen S, Linke A, Conradi K, Erbs S, Kluge R, Kendziorra K, Sabri O, Sick P, Schuler G. Circulation. 2004 Mar 23;109(11):1371-8. | Excluded : unconventional treatment |
| 81 | Three-year outcome after coronary stenting versus bypass surgery for the treatment of multivessel disease. Legrand VM, Serruys PW, Unger F, van Hout BA, Vrolix MC, Fransen GM, Nielsen TT, Paulsen PK, Gomes RS, de Queiroz e Melo JM, Neves JP, Lindeboom W, Backx B; Arterial Revascularization Therapy Study (ARTS) Investigators. Circulation. 2004 Mar 9;109(9):1114-20. | selected |
| 82 | Cost analysis of aprotinin for coronary artery bypass patients: analysis of the randomized trials. Smith PK, Datta SK, Muhlbaier LH, Samsa G, Nadel A, Lipscomb J. Ann Thorac Surg. 2004 Feb;77(2):635-42 | Excluded : other intervention was studied |
| 83 | Evaluation of outcome and cost-effectiveness using an FDG PET-guided approach to management of patients with coronary disease and severe left ventricular dysfunction (PARR-2): rationale, design, and methods. Beanlands R, Nichol G, Ruddy TD, deKemp RA, Hendry P, Humen D, Racine N, Ross H, Benard F, Coates G, Iwanochko RM, Fallen E, Wells G; PARR-2 Investigators. Control Clin Trials. 2003 Dec;24(6):776-94. | Excluded : other intervention was studied |
| 84 | Cost-effectiveness of coronary stenting and abciximab for patients with acute myocardial infarction: results from the CADILLAC (Controlled Abciximab and Device Investigation to Lower Late Angioplasty Complications) trial. Bakhai A, Stone GW, Grines CL, Murphy SA, Githiora L, Berezin RH, Cox DA, Stuckey T, Griffin JJ, Tcheng JE, Cohen DJ; CADILLAC Investigators. Circulation. 2003 Dec 9;108(23):2857-63. | Excluded : other intervention was studied |
| 85 | Cost-effectiveness of bypass surgery versus stenting in patients with multivessel coronary artery disease. Yock CA, Boothroyd DB, Owens DK, Garber AM, Hlatky MA. Am J Med. 2003 Oct 1;115(5):382-9. | Excluded : economic model |
| 86 | Relative cost comparison of treatments for coronary artery disease: the First Year Follow-Up of MASS II Study. Favarato D, Hueb W, Gersh BJ, Soares PR, Cesar LA, da Luz PL, Oliveira SA, Ramires JA; First Year Follow-Up of MASS II Study. Circulation. 2003 Sep 9;108 Suppl 1:II21-3. | selected |
| 87 | Continued improvement of clinical outcome and cost effectiveness following intravascular ultrasound guided PCI: insights from a prospective, randomised study. Gaster AL, Slothuus Skjoldborg U, Larsen J, Korsholm L, von Birgelen C, Jensen S, Thayssen P, Pedersen KE, Haghfelt TH. Heart. 2003 Sep;89(9):1043-9. | Excluded : other intervention was studied |
| 88 | Benefits of off-pump bypass on neurologic and clinical morbidity: a prospective randomized trial. Lee JD, Lee SJ, Tsushima WT, Yamauchi H, Lau WT, Popper J, Stein A, Johnson D, Lee D, Petrovitch H, Dang CR. Ann Thorac Surg. 2003 Jul;76(1):18-25; discussion 25-6. | Excluded : unconventional treatment |
| 89 | Comparison of analytic approaches for the economic evaluation of new technologies alongside multicenter clinical trials. Taira DA, Seto TB, Siegrist R, Cosgrove R, Berezin R, Cohen DJ. Am Heart J. 2003 Mar;145(3):452-8. | Excluded : it was a protocol |
| 90 | Small molecule glycoprotein IIb/IIIa receptor inhibitors as upstream therapy in acute coronary syndromes: insights from the TACTICS TIMI-18 trial. Cannon CP. J Am Coll Cardiol. 2003 Feb 19;41(4 Suppl S):43S-48S | Excluded : population with different inclusion criteria (acute coronary syndrom) |
| 91 | Direct coronary stent implantation does not reduce the incidence of in-stent restenosis or major adverse cardiac events: six month results of a randomized trial. IJsselmuiden AJ, Serruys PW, Scholte A, Kiemeneij F, Slagboom T, vd Wieken LR, Tangelder GJ, Laarman GJ. Eur Heart J. 2003 Mar;24(5):421-9. | Excluded : follow up doesn't correspond to (less than 1 year or different than 1 and/or 3 years) |
| 92 | Direct stenting with the Bx VELOCITY balloon-expandable stent mounted on the Raptor rapid exchange delivery system versus predilatation in a European randomized Trial: the VELVET trial. Serruys PW, IJsselmuiden S, Hout Bv, Vermeersch P, Bramucci E, Legrand V, Pieper M, Antoniucci D, Gomes RS, Macaya C, Boekstegers P, Lindeboom W; VELVET investigators. Int J Cardiovasc Intervent. 2003;5(1):17-26. | Excluded : follow up doesn't correspond to (less than 1 year or different than 1 and/or 3 years) |
| 93 | Randomized comparison of direct and provisional stenting in de novo coronary artery lesions: the RADICAL study. Weber F, Schneider H, Warzok F, Petzsch M, von Knorre GH, Nienaber CA. Z Kardiol. 2003 Feb;92(2):173-81. | Excluded : follow up doesn't correspond to (less than 1 year or different than 1 and/or 3 years) |
| 94 | Intravascular ultrasound-guided balloon angioplasty compared with stent: immediate and 6-month results of the multicenter, randomized Balloon Equivalent to Stent Study (BEST). Schiele F, Meneveau N, Gilard M, Boschat J, Commeau P, Ming LP, Sewoke P, Seronde MF, Mercier M, Gupta S, Bassand JP; Balloon Equivalent to Stent Study. Circulation. 2003 Feb 4;107(4):545-51. | Excluded : follow up doesn't correspond to (less than 1 year or different than 1 and/or 3 years) |
| 95 | A comparison of on-pump and off-pump coronary bypass surgery in low-risk patients. Nathoe HM, van Dijk D, Jansen EW, Suyker WJ, Diephuis JC, van Boven WJ, de la Rivière AB, Borst C, Kalkman CJ, Grobbee DE, Buskens E, de Jaegere PP; Octopus Study Group. N Engl J Med. 2003 Jan 30;348(5):394-402. | Excluded : one treatment studied was not conventionnal |
| 96 | Quality of life and time trade-off utility measures in patients with coronary artery disease. Melsop KA, Boothroyd DB, Hlatky MA. Am Heart J. 2003 Jan;145(1):36-41 | Excluded : incomplete data (no economic data available at 1 and/or 3 years) |
| 97 | Major outcomes in high-risk hypertensive patients randomized to angiotensin-converting enzyme inhibitor or calcium channel blocker vs diuretic: The Antihypertensive and Lipid-Lowering Treatment to Prevent Heart Attack Trial (ALLHAT). ALLHAT Officers and Coordinators for the ALLHAT Collaborative Research Group. The Antihypertensive and Lipid-Lowering Treatment to Prevent Heart Attack Trial. JAMA. 2002 Dec 18;288(23):2981-97. | Excluded : other intervention was studied |
| 98 | Direct coronary stenting versus predilatation followed by stent placement. Brueck M, Scheinert D, Wortmann A, Bremer J, von Korn H, Klinghammer L, Kramer W, Flachskampf FA, Daniel WG, Ludwig J. Am J Cardiol. 2002 Dec 1;90(11):1187-92. | Excluded : follow up doesn't correspond to (less than 1 year or different than 1 and/or 3 years) |
| 99 | Clinical and angiographic outcome after conventional angioplasty with optional stent implantation compared with direct stenting without predilatation. Miketic S, Carlsson J, Tebbe U. Heart. 2002 Dec;88(6):622-6. | Excluded : follow up doesn't correspond to (less than 1 year or different than 1 and/or 3 years) |
| 100 | Comparison of costs and safety of a suture-mediated closure device with conventional manual compression after coronary artery interventions. Rickli H, Unterweger M, Sütsch G, Brunner-La Rocca HP, Sagmeister M, Ammann P, Amann FW. Catheter Cardiovasc Interv. 2002 Nov;57(3):297-302. | Excluded : other intervention was studied |
| 101 | Impact of intracoronary ultrasound guidance on long-term outcome of percutaneous coronary interventions in diabetics--insights from the randomized SIPS trial. Mueller C, Mc Hodgson JB, Brutsche M, Perruchoud AP, Marsch S, Hunziker P, Buettner HJ. Swiss Med Wkly. 2002 Jun 1;132(21-22):279-84. | Excluded : population with different inclusion criteria (diabetes mellitus) |
| 102 | Is direct coronary stenting the best strategy for long-term outcome? Results of the multicentric randomized benefit evaluation of direct coronary stenting (BET) study. Elbaz M, El Mokhtar E, Khalifé K, Citron B, Izaaz K, Hamon M, Juliard JM, Leclercq F, Fourcade J, Lipiecki J, Sabatier R, Boulet V, Rinaldi JP, Mourali S, Fatouch M, Asmar A, Steg PG, Puel J, Carrié D. Am Heart J. 2002 Oct;144(4):E7. | Excluded : incomplete data (no economic data available at 1 and/or 3 years) |
| 103 | Prospective analysis of creatine kinase muscle-brain fraction and comparison with troponin T to predict cardiac risk and benefit of an invasive strategy in patients with non-ST-elevation acute coronary syndromes. Kleiman NS, Lakkis N, Cannon CP, Murphy SA, DiBattiste PM, Demopoulos LA, Weintraub WS, Braunwald E; TACTICS-TIMI 18 Investigators. J Am Coll Cardiol. 2002 Sep 18;40(6):1044-50. | Excluded : population with different inclusion criteria (acute coronary syndrom) |
| 104 | Resource utilization, cost, and health status impacts of coronary stent versus "optimal" percutaneous coronary angioplasty: results from the OPUS-I trial. Neil N, Ramsey SD, Cohen DJ, Every NR, Spertus JA, Weaver WD; OPUS-I Investigators. J Interv Cardiol. 2002 Aug;15(4):249-55. | Excluded : follow up doesn't correspond to (less than 1 year or different than 1 and/or 3 years) |
| 105 | Efficacy and safety of multivessel percutaneous revascularization and tirofiban therapy in patients with acute coronary syndromes. Brener SJ, Murphy SA, Gibson CM, DiBattiste PM, Demopoulos LA, Cannon CP; TACTICS-TIMI 18 Investigators. Treat Angina with Aggrastat and Determine Cost of Therapy with an Invasive or Conservative Strategy-Thrombosis in Myocardial Infarction. Am J Cardiol. 2002 Sep 15;90(6):631-3. | Excluded : population with different inclusion criteria (acute coronary syndrom) |
| 106 | Randomized comparison of success and adverse event rates and cost effectiveness of one long versus two short stents for treatment of long coronary narrowings. Hoffmann R, Herrmann G, Silber S, Braun P, Werner GS, Hennen B, Rupprecht H, vom Dahl J, Hanrath P; IMPact Upon Long Lesion StEnting Study Group. Am J Cardiol. 2002 Sep 1;90(5):460-4. | Excluded : follow up doesn't correspond to (less than 1 year or different than 1 and/or 3 years) |
| 107 | Coronary angioplasty versus medical therapy for angina. Health service costs based on the second Randomized Intervention Treatment of Angina (RITA-2) trial. Sculpher M, Smith D, Clayton T, Henderson R, Buxton M, Pocock S, Chamberlain D; Randomized Intervention Treatment of Angina (RITA-2) trial. Eur Heart J. 2002 Aug;23(16):1291-1300. | selected |
| 108 | More reliable oximetry reduces the frequency of arterial blood gas analyses and hastens oxygen weaning after cardiac surgery: a prospective, randomized trial of the clinical impact of a new technology. Durbin CG Jr, Rostow SK. Crit Care Med. 2002 Aug;30(8):1735-40. | Excluded : other intervention was studied |
| 109 | Cost-effectiveness of gamma radiation for treatment of in-stent restenosis: results from the Gamma-1 trial. Cohen DJ, Cosgrove RS, Berezin RH, Teirstein PS, Leon MB, Kuntz RE; Gamma-1 Investigators. Circulation. 2002 Aug 6;106(6):691-7. | Excluded : population with different inclusion criteria |
| 110 | A pharmacoeconomic evaluation of results from the Coronary Angioplasty Amlodipine Restenosis Study (CAPARES) in Norway and Canada. Thaulow E, Jorgensen B, Doyle JJ, Casciano R, Casciano J, Kopp Z, Arikian S, Kim R. Int J Cardiol. 2002 Jul;84(1):23-30 | Excluded : economic model |
| 111 | Could direct stenting reduce no-reflow in acute coronary syndromes? A randomized pilot study. Sabatier R, Hamon M, Zhao QM, Burzotta F, Lecluse E, Valette B, Grollier G. Am Heart J. 2002 Jun;143(6):1027-32. | Excluded : population with different inclusion criteria (acute coronary syndrom) |
| 112 | Bypass surgery versus stenting for the treatment of multivessel disease in patients with unstable angina compared with stable angina. de Feyter PJ, Serruys PW, Unger F, Beyar R, de Valk V, Milo S, Simon R, Regensburger D, Crean PA, McGovern E, van den Heuvel P, van Cauwelaert C, Penn I, Tyers GF, Lindeboom W. Circulation. 2002 May 21;105(20):2367-72. | Excluded : doubloon (data used in another reference) |
| 113 | Impact of clinical syndrome acuity on the differential response to 2 glycoprotein IIb/IIIa inhibitors in patients undergoing coronary stenting: the TARGET Trial. Stone GW, Moliterno DJ, Bertrand M, Neumann FJ, Herrmann HC, Powers ER, Grines CL, Moses JW, Cohen DJ, Cohen EA, Cohen M, Wolski K, DiBattiste PM, Topol EJ. Circulation. 2002 May 21;105(20):2347-54. | Excluded : population with different inclusion criteria (acute coronary syndrom) |
| 114 | Glycoprotein IIb-IIIa inhibition with abciximab and postprocedural risk assessment: lessons from the evaluation of platelet IIb/IIIa inhibitor for stenting trial and implication for ad hoc use of glycoprotein IIb-IIIa antagonists. Mazur W, Kaluza GL, Sapp S, Balog C, Topol EJ, Mark DB, Ellis SG, Kereiakes DJ, Lincoff AM, Kleiman NS. Am Heart J. 2002 Apr;143(4):594-601. | Excluded : follow up doesn't correspond to (less than 1 year or different than 1 and/or 3 years) |
| 115 | Cost-effectiveness of a conservative, ischemia-guided management strategy after non-Q-wave myocardial infarction: results of a randomized trial. Barnett PG, Chen S, Boden WE, Chow B, Every NR, Lavori PW, Hlatky MA. Circulation. 2002 Feb 12;105(6):680-4. | Excluded : population with different inclusion criteria (acute coronary syndrom) |
| 116 | Cost effectiveness of eptifibatide in acute coronary syndromes; an economic analysis of Western European patients enrolled in the PURSUIT trial. The Platelet IIa/IIb in unstable Angina: Receptor Suppression Using Integrilin Therapy. Brown RE, Henderson RA, Koster D, Hutton J, Simoons ML. Eur Heart J. 2002 Jan;23(1):50-8. | Excluded : population with different inclusion criteria |
| 117 | Cost-effectiveness of an invasive strategy in unstable coronary artery disease; results from the FRISC II invasive trial. The Fast Revascularisation during InStability in Coronary artery disease. Janzon M, Levin LA, Swahn E. Eur Heart J. 2002 Jan;23(1):31-40. | Excluded : follow up doesn't correspond to (less than 1 year or different than 1 and/or 3 years) |
| 118 | A randomized trial of stenting with or without balloon predilation for the treatment of coronary artery disease. Kovar LI, Monrad ES, Sherman W, Kunchithapatham S, Ravi KL, Gotsis W, Silverman G, Brown DL. Am Heart J. 2001 Nov;142(5):E9. | Excluded : follow up doesn't correspond to (less than 1 year or different than 1 and/or 3 years) |
| 119 | Early outcome after off-pump versus on-pump coronary bypass surgery: results from a randomized study. van Dijk D, Nierich AP, Jansen EW, Nathoe HM, Suyker WJ, Diephuis JC, van Boven WJ, Borst C, Buskens E, Grobbee DE, Robles De Medina EO, de Jaegere PP; Octopus Study Group. Circulation. 2001 Oct 9;104(15):1761-6. | Excluded : one treatment studied was not conventionnal |
| 120 | Direct coronary stenting without balloon or device pretreatment: acute success and long-term results. Stys T, Lawson WE, Liuzzo JP, Hanif B, Bragg L, Cohn PF. Catheter Cardiovasc Interv. 2001 Oct;54(2):158-63. | Excluded : follow up doesn't correspond to (less than 1 year or different than 1 and/or 3 years) |
| 121 | Economic assessment of rheolytic thrombectomy versus intracoronary urokinase for treatment of extensive intracoronary thrombus: Results from a randomized clinical trial. Cohen DJ, Ramee S, Baim DS, Sharma S, Carrozza JP, Cosgrove R, Jones N, Berezin RH, Cutlip DE, Ho KK, Kuntz RE; Vein Graft AngioJet Study (VeGAS) 2 Investigators. Am Heart J. 2001 Oct;142(4):648-56. | Excluded : other intervention was studied |
| 122 | Effectiveness of bi-atrial pacing for reducing atrial fibrillation after coronary artery bypass graft surgery. Gerstenfeld EP, Khoo M, Martin RC, Cook JR, Lancey R, Rofino K, Vander Salm TJ, Mittleman RS. J Interv Card Electrophysiol. 2001 Sep;5(3):275-83. | Excluded : other intervention was studied |
| 123 | Clinical and economic impact of diabetes mellitus on percutaneous and surgical treatment of multivessel coronary disease patients: insights from the Arterial Revascularization Therapy Study (ARTS) trial. Abizaid A, Costa MA, Centemero M, Abizaid AS, Legrand VM, Limet RV, Schuler G, Mohr FW, Lindeboom W, Sousa AG, Sousa JE, van Hout B, Hugenholtz PG, Unger F, Serruys PW; Arterial Revascularization Therapy Study Group. Circulation. 2001 Jul 31;104(5):533-8. | Excluded : population with different inclusion criteria (only patients with diabetes mellitus) |
| 124 | Arterial Revascularization Therapies Study (ARTS). [No authors listed] Indian Heart J. 2001 Mar-Apr;53(2):239. | Excluded : it was a commentary |
| 125 | Comparison of early invasive and conservative strategies in patients with unstable coronary syndromes treated with the glycoprotein IIb/IIIa inhibitor tirofiban. Cannon CP, Weintraub WS, Demopoulos LA, Vicari R, Frey MJ, Lakkis N, Neumann FJ, Robertson DH, DeLucca PT, DiBattiste PM, Gibson CM, Braunwald E; TACTICS (Treat Angina with Aggrastat and Determine Cost of Therapy with an Invasive or Conservative Strategy)--Thrombolysis in Myocardial Infarction 18 Investigators. N Engl J Med. 2001 Jun 21;344(25):1879-87. | Excluded : follow up doesn't correspond to (less than 1 year or different than 1 and/or 3 years) |
| 126 | Cost-effectiveness analysis of intravascular ultrasound guided percutaneous coronary intervention versus conventional percutaneous coronary intervention. Gaster AL, Slothuus U, Larsen J, Thayssen P, Haghfelt T. Scand Cardiovasc J. 2001 Mar;35(2):80-5. | Excluded : one treatment studied was not conventionnal |
| 127 | Long term outcome and cost-effectiveness of stenting versus balloon angioplasty for acute myocardial infarction. Suryapranata H, Ottervanger JP, Nibbering E, van 't Hof AW, Hoorntje JC, de Boer MJ, Al MJ, Zijlstra F. Heart. 2001 Jun;85(6):667-71. | Excluded : population with different inclusion criteria (acute myocardial infarction) |
| 128 | Comparison of coronary-artery bypass surgery and stenting for the treatment of multivessel disease. Serruys PW, Unger F, Sousa JE, Jatene A, Bonnier HJ, Schönberger JP, Buller N, Bonser R, van den Brand MJ, van Herwerden LA, Morel MA, van Hout BA; Arterial Revascularization Therapies Study Group. N Engl J Med. 2001 Apr 12;344(15):1117-24. | selected |
| 129 | Reduced postoperative blood loss and transfusion requirement after beating-heart coronary operations: a prospective randomized study. Ascione R, Williams S, Lloyd CT, Sundaramoorthi T, Pitsis AA, Angelini GD. J Thorac Cardiovasc Surg. 2001 Apr;121(4):689-96. | Excluded : one treatment studied was not conventionnal |
| 130 | Comparison of direct coronary stenting with and without balloon predilatation in patients with stable angina pectoris. BET (Benefit Evaluation of Direct Coronary Stenting) Study Group. Carrié D, Khalifé K, Citron B, Izaaz K, Hamon M, Juiliard JM, Leclercq F, Fourcade J, Lipiecki J, Sabatier R, Boulet V, Rinaldi JP, Mourali S, Fatouch M, El Mokhtar E, Aboujaoudé G, Elbaz M, Grolleau R, Steg PG, Puel J; Benefit Evaluation of Direct Coronary Stenting Study Group. Am J Cardiol. 2001 Mar 15;87(6):693-8. | Excluded : follow up doesn't correspond to (less than 1 year or different than 1 and/or 3 years) |
| 131 | Outcomes of noncardiac surgery after coronary bypass surgery or coronary angioplasty in the Bypass Angioplasty Revascularization Investigation (BARI). Hassan SA, Hlatky MA, Boothroyd DB, Winston C, Mark DB, Brooks MM, Eagle KA. Am J Med. 2001 Mar;110(4):260-6. | Excluded : incomplete data (no economic data available at 1 and/or 3 years) |
| 132 | Comparative 30-day economic and clinical outcomes of platelet glycoprotein IIb/IIIa inhibitor use during elective percutaneous coronary intervention: Prairie ReoPro versus Integrilin Cost Evaluation (PRICE) Trial. PRICE Investigators. Am Heart J. 2001 Mar;141(3):402-9. | Excluded : follow up doesn't correspond to (less than 1 year or different than 1 and/or 3 years) |
| 133 | Clinical trials of coronary revascularization: coronary angioplasty vs. coronary bypass grafting. Bourassa MG. Curr Opin Cardiol. 2000 Jul;15(4):281-6 | Excluded : it was a review |
| 134 | Effect of lovastatin on cardiovascular resource utilization and costs in the Air Force/Texas Coronary Atherosclerosis Prevention Study (AFCAPS/TexCAPS). AFCAPS/TexCAPS Research Group. Gotto AM Jr, Boccuzzi SJ, Cook JR, Alexander CM, Roehm JB, Meyer GS, Clearfield M, Weis S, Whitney E. Am J Cardiol. 2000 Dec 1;86(11):1176-81. | Excluded : other intervention was studied |
| 135 | Is provisional stenting the effective option? The WIDEST study (Wiktor stent in de novo stenosis). Widest Trial Investigators' Group. Fluck DS, Chenu P, Mills P, Davies A, Street J, Paul E, Balcon R, Layton CA. Heart. 2000 Nov;84(5):522-8. | Excluded : incomplete data (no economic data available at 1 and/or 3 years) |
| 136 | Costs of revascularization over eight years in the randomized and eligible patients in the Emory Angioplasty versus Surgery Trial (EAST). Weintraub WS, Becker ER, Mauldin PD, Culler S, Kosinski AS, King SB 3rd. Am J Cardiol. 2000 Oct 1;86(7):747-52. | Excluded : incomplete data (Myocardial infarction rate was not available) |
| 137 | Projected long-term costs of coronary stenting in multivessel coronary disease based on the experience of the Bypass Angioplasty Revascularization Investigation (BARI). Yock CA, Boothroyd DB, Owens DK, Winston C, Hlatky MA. Am Heart J. 2000 Oct;140(4):556-64. | Excluded : economic model |
| 138 | Outcome at 1 year after an invasive compared with a non-invasive strategy in unstable coronary-artery disease: the FRISC II invasive randomised trial. FRISC II Investigators. Fast Revascularisation during Instability in Coronary artery disease. Wallentin L, Lagerqvist B, Husted S, Kontny F, Ståhle E, Swahn E. Lancet. 2000 Jul 1;356(9223):9-16. | Excluded : population with different inclusion criteria (acute coronary syndrom) |
| 139 | Optimum percutaneous transluminal coronary angioplasty compared with routine stent strategy trial (OPUS-1): a randomised trial. Weaver WD, Reisman MA, Griffin JJ, Buller CE, Leimgruber PP, Henry T, D'Haem C, Clark VL, Martin JS, Cohen DJ, Neil N, Every NR. Lancet. 2000 Jun 24;355(9222):2199-203. | Excluded : follow up doesn't correspond to (less than 1 year or different than 1 and/or 3 years) |
| 140 | A randomised controlled trial of Prostar Plus for haemostasis in patients after coronary angioplasty. Noguchi T, Miyazaki S, Yasuda S, Baba T, Sumida H, Morii I, Daikoku S, Goto Y, Nonogi H. Eur J Vasc Endovasc Surg. 2000 May;19(5):451-5. | Excluded : other intervention was studied |
| 141 | Effective prevention of atrial fibrillation by continuous atrial overdrive pacing after coronary artery bypass surgery. Blommaert D, Gonzalez M, Mucumbitsi J, Gurné O, Evrard P, Buche M, Louagie Y, Eucher P, Jamart J, Installé E, De Roy L. J Am Coll Cardiol. 2000 May;35(6):1411-5. | Excluded : other intervention was studied |
| 142 | Outcomes at 1 year and economic implications of platelet glycoprotein IIb/IIIa blockade in patients undergoing coronary stenting: results from a multicentre randomised trial. EPISTENT Investigators. Evaluation of Platelet IIb/IIIa Inhibitor for Stenting. Topol EJ, Mark DB, Lincoff AM, Cohen E, Burton J, Kleiman N, Talley D, Sapp S, Booth J, Cabot CF, Anderson KM, Califf RM. Lancet. 1999 Dec 11;354(9195):2019-24. | Excluded : population with different inclusion criteria (acute coronary syndrom) |
| 143 | Suture closure of femoral arterial puncture sites after coronary angioplasty followed by same-day discharge. Carere RG, Webb JG, Buller CE, Wilson M, Rahman T, Spinelli J, Anis AH. Am Heart J. 2000 Jan;139(1 Pt 1):52-8. | Excluded : other intervention was studied |
| 144 | Cost estimates for treatment of cardiac ischemia (from the Asymptomatic Cardiac Ischemia Pilot [ACIP] study). Pepine CJ, Mark DB, Bourassa MG, Chaitman BR, Davies RF, Knatterud GL, Forman S, Pratt CM, Sopko G, Conti CR. Am J Cardiol. 1999 Dec 1;84(11):1311-6. | Excluded : incomplete data (study with 3 arms which 2 for drugs and one revascularization) |
| 145 | Clinical benefits of endoscopic vein harvesting in patients with risk factors for saphenectomy wound infections undergoing coronary artery bypass grafting. Carpino PA, Khabbaz KR, Bojar RM, Rastegar H, Warner KG, Murphy RE, Payne DD. J Thorac Cardiovasc Surg. 2000 Jan;119(1):69-75. | Excluded : other intervention |
| 146 | Minimally invasive saphenous vein harvesting: is there an improvement of the results with the endoscopic approach? Isgro F, Weisse U, Voss B, Kiessling AH, Saggau W. Eur J Cardiothorac Surg. 1999 Nov;16 Suppl 2:S58-60. | Excluded : other intervention |
| 147 | A randomized trial of endoscopic versus open saphenous vein harvest in coronary bypass surgery. Puskas JD, Wright CE, Miller PK, Anderson TE, Gott JP, Brown WM 3rd, Guyton RA. Ann Thorac Surg. 1999 Oct;68(4):1509-12. | Excluded : other intervention |
| 148 | Economic impact of GPIIB/IIIA blockade after high-risk angioplasty: results from the RESTORE trial. Randomized Efficacy Study of Tirofiban for Outcomes and Restenosis. Weintraub WS, Culler S, Boccuzzi SJ, Cook JR, Kosinski AS, Cohen DJ, Burnette J. J Am Coll Cardiol. 1999 Oct;34(4):1061-6. | Excluded : follow up doesn't correspond to (less than 1 year or different than 1 and/or 3 years) |
| 149 | Optimal timing of preoperative intraaortic balloon pump support in high-risk coronary patients. Christenson JT, Simonet F, Badel P, Schmuziger M. Ann Thorac Surg. 1999 Sep;68(3):934-9. | Excluded : unconventional treatment |
| 150 | Synthetic protein treated versus heparin coated cardiopulmonary bypass surfaces: similar clinical results and minor biochemical differences. Wimmer-Greinecker G, Matheis G, Martens S, Oremek G, Abdel-Rahman U, Moritz A. Eur J Cardiothorac Surg. 1999 Aug;16(2):211-7. | Excluded : other intervention |
| 151 | Clinical correlates of the initial and long-term cost of coronary bypass surgery and coronary angioplasty. Hlatky MA, Boothroyd DB, Brooks MM, Winston C, Rosen A, Rogers WJ, Reeder GS, Smith HC, Ryan TJ, Pitt B, Whitlow PL, Wiens RD, Mark DB. Am Heart J. 1999 Aug;138(2 Pt 1):376-83. | Excluded : follow up doesn't correspond to (less than 1 year or different than 1 and/or 3 years) |
| 152 | Emerging treatment of acute coronary syndromes with platelet glycoprotein IIB/IIIA inhibitors. Roe MT, Moliterno DJ. J Thromb Thrombolysis. 1999 Jun;7(3):247-57. | Excluded : population with different inclusion criteria (acute coronary syndrom) |
| 153 | Prophylactic coronary artery revascularization for elective vascular surgery: study design. Veterans Affairs Cooperative Study Group on Coronary Artery Revascularization Prophylaxis for Elective Vascular Surgery. McFalls EO, Ward HB, Krupski WC, Goldman S, Littooy F, Eagle K, Nyman JA, Moritz T, McNabb S, Henderson WG. Control Clin Trials. 1999 Jun;20(3):297-308. | Excluded : it was a protocol |
| 154 | A two-center study evaluating the hemodynamic and pharmacodynamic effects of cisatracurium and vecuronium in patients undergoing coronary artery bypass surgery. Searle NR, Thomson I, Dupont C, Cannon JE, Roy M, Rosenbloom M, Gagnon L, Carrier M. J Cardiothorac Vasc Anesth. 1999 Feb;13(1):20-5. | Excluded : other intervention |
| 155 | Pre-operative short-term pulmonary rehabilitation for patients of chronic obstructive pulmonary disease undergoing coronary artery bypass graft surgery. Rajendran AJ, Pandurangi UM, Murali R, Gomathi S, Vijayan VK, Cherian KM. Indian Heart J. 1998 Sep-Oct;50(5):531-4. | Excluded : other intervention |
| 156 | Optimal coronary balloon angioplasty with provisional stenting versus primary stent (OCBAS): immediate and long-term follow-up results. Rodríguez A, Ayala F, Bernardi V, Santaera O, Marchand E, Pardiñas C, Mauvecin C, Vogel D, Harrell LC, Palacios IF. J Am Coll Cardiol. 1998 Nov;32(5):1351-7. | Excluded : follow up doesn't correspond to (less than 1 year or different than 1 and/or 3 years) |
| 157 | Long-term results of RITA-1 trial: clinical and cost comparisons of coronary angioplasty and coronary-artery bypass grafting. Randomised Intervention Treatment of Angina. Henderson RA, Pocock SJ, Sharp SJ, Nanchahal K, Sculpher MJ, Buxton MJ, Hampton JR. Lancet. 1998 Oct 31;352(9138):1419-25. | Excluded : follow up doesn't correspond to (less than 1 year or different than 1 and/or 3 years) |
| 158 | Randomised comparison of implantation of heparin-coated stents with balloon angioplasty in selected patients with coronary artery disease (Benestent II) Serruys PW, van Hout B, Bonnier H, Legrand V, Garcia E, Macaya C, Sousa E, van der Giessen W, Colombo A, Seabra-Gomes R, Kiemeneij F, Ruygrok P, Ormiston J, Emanuelsson H, Fajadet J, Haude M, Klugmann S, Morel MA. Lancet. 1998 Aug 29;352(9129):673-81. | selected |
| 159 | Prolonged heparin after uncomplicated coronary interventions: a prospective, randomized trial. Garachemani AR, Kaufmann U, Fleisch M, Meier B. Am Heart J. 1998 Aug;136(2):352-6. | Excluded : follow up doesn't correspond to (less than 1 year or different than 1 and/or 3 years) |
| 160 | Safety and cost-effectiveness of early discharge after primary angioplasty in low risk patients with acute myocardial infarction. PAMI-II Investigators. Primary Angioplasty in Myocardial Infarction. Grines CL, Marsalese DL, Brodie B, Griffin J, Donohue B, Costantini CR, Balestrini C, Stone G, Wharton T, Esente P, Spain M, Moses J, Nobuyoshi M, Ayres M, Jones D, Mason D, Sachs D, Grines LL, O'Neill W. J Am Coll Cardiol. 1998 Apr;31(5):967-72. | Excluded : population with different inclusion criteria (acute myocardial infarction) |
| 161 | Costs and effects in therapy for acute coronary syndromes: the case of abciximab in high-risk patients undergoing percutaneous transluminal coronary angioplasty in the EPIC study. Evaluation of 7E3 for the Prevention of Ischemic Complications. van Hout BA, Bowman L, Zelinger DJ, Simoons ML. Am Heart J. 1998 Apr;135(4):S98-106. | Excluded : population with different inclusion criteria (acute coronary syndrom) |
| 162 | Influence of a randomized clinical trial on practice by participating investigators: lessons from the Coronary Angioplasty Versus Excisional Atherectomy Trial (CAVEAT). CAVEAT I and II Investigators. Omoigui NA, Silver MJ, Rybicki LA, Rosenthal M, Berdan LG, Pieper K, King SV, Califf RM, Topol EJ. J Am Coll Cardiol. 1998 Feb;31(2):265-72. | Excluded : other intervention was studied |
| 163 | Compliance and adverse event withdrawal: their impact on the West of Scotland Coronary Prevention Study. [No authors listed] Eur Heart J. 1997 Nov;18(11):1718-24. | Excluded : other intervention |
| 164 | Rationale for low-molecular weight heparin in coronary stenting. Zidar JP. Am Heart J. 1997 Nov;134(5 Pt 2):S81-7. | Excluded : other intervention |
| 165 | Preoperative intraaortic balloon pump enhances cardiac performance and improves the outcome of redo CABG. Christenson JT, Badel P, Simonet F, Schmuziger M. Ann Thorac Surg. 1997 Nov;64(5):1237-44. | Excluded : other intervention |
| 166 | The effect of preoperative intra-aortic balloon pump support in patients with coronary artery disease, poor left-ventricular function (LVEF < 40%), and hypertensive LV hypertrophy. Christenson JT, Simonet F, Badel P, Schmuziger M. Thorac Cardiovasc Surg. 1997 Apr;45(2):60-4. | Excluded : other intervention was studied |
| 167 | Hemostatic effects of three colloid plasma substitutes for priming solution in cardiopulmonary bypass. Tigchelaar I, Gallandat Huet RC, Korsten J, Boonstra PW, van Oeveren W. Eur J Cardiothorac Surg. 1997 Apr;11(4):626-32. | Excluded : other intervention |
| 168 | Expansion of Wiktor stents by oversizing versus high-pressure dilatation: a randomized, intracoronary ultrasound-controlled study. Buchwald AB, Werner GS, Möller K, Unterberg C. Am Heart J. 1997 Feb;133(2):190-6. | Excluded : follow up doesn't correspond to (less than 1 year or different than 1 and/or 3 years) |
| 169 | Medical care costs and quality of life after randomization to coronary angioplasty or coronary bypass surgery. Bypass Angioplasty Revascularization Investigation (BARI) Investigators. Hlatky MA, Rogers WJ, Johnstone I, Boothroyd D, Brooks MM, Pitt B, Reeder G, Ryan T, Smith H, Whitlow P, Wiens R, Mark DB. N Engl J Med. 1997 Jan 9;336(2):92-9. | selected |
| 170 | Cost effectiveness of stent implantation versus PTCA: the BENESTENT experience. Van Hout BA, van der Woude T, de Jaegere PP, van den Brand M, van Es GA, Serruys PW, Morel MA. Semin Interv Cardiol. 1996 Dec;1(4):263-8. | Excluded : follow up doesn't correspond to (less than 1 year or different than 1 and/or 3 years) |
| 171 | Plateletpheresis before redo CABG diminishes excessive blood transfusion. Christenson JT, Reuse J, Badel P, Simonet F, Schmuziger M. Ann Thorac Surg. 1996 Nov;62(5):1373-8; discussion 1378-9. | Excluded : other intervention was studied |
| 172 | Increased risk of non-Q wave myocardial infarction after directional atherectomy is platelet dependent: evidence from the EPIC trial. Evaluation of c7E3 for the Prevention of Ischemic Complications. Lefkovits J, Blankenship JC, Anderson KM, Stoner GL, Talley JD, Worley SJ, Weisman HF, Califf RM, Topol EJ. J Am Coll Cardiol. 1996 Oct;28(4):849-55. | Excluded : unconventional treatment |
| 173 | Cholesterol lowering and the use of healthcare resources. Results of the Scandinavian Simvastatin Survival Study. Pedersen TR, Kjekshus J, Berg K, Olsson AG, Wilhelmsen L, Wedel H, Pyörälä K, Miettinen T, Haghfelt T, Faergeman O, Thorgeirsson G, Jönsson B, Schwartz JS. Circulation. 1996 May 15;93(10):1796-802. | Excluded : 2 treatments identical (medical therapy) |
| 174 | Three-year follow-up of the Argentine Randomized Trial of Percutaneous Transluminal Coronary Angioplasty Versus Coronary Artery Bypass Surgery in Multivessel Disease (ERACI). Rodriguez A, Mele E, Peyregne E, Bullon F, Perez-Baliño N, Liprandi MI, Palacios IF. J Am Coll Cardiol. 1996 Apr;27(5):1178-84. | selected |
| 175 | Mortality, reinfarction, left ventricular ejection fraction and costs following reperfusion therapies for acute myocardial infarction. Zijlstra F, de Boer MJ, Beukema WP, Liem AL, Reiffers S, Huysmans D, Hoorntje JC, Suryapranata H, Simoons ML. Eur Heart J. 1996 Mar;17(3):382-7. | Excluded : population with different inclusion criteria (acute myocardial infarction) |
| 176 | Costs of coronary restenosis (Lovastatin Restenosis Trial). Gilbert SP, Weintraub WS, Talley JD, Boccuzzi SJ. Am J Cardiol. 1996 Jan 15;77(2):196-9. | Excluded : population with different inclusion criteria |
| 177 | Autologous platelet sequestration in patients undergoing coronary artery bypass grafting. Christenson JT, Reuse J, Badel P, Nowicki B, Simonet F, Schmuziger M. Eur J Cardiothorac Surg. 1996;10(12):1083-9. | Excluded : other intervention was studied |
| 178 | A comparison of the costs of and quality of life after coronary angioplasty or coronary surgery for multivessel coronary artery disease. Results from the Emory Angioplasty Versus Surgery Trial (EAST). Weintraub WS, Mauldin PD, Becker E, Kosinski AS, King SB 3rd. Circulation. 1995 Nov 15;92(10):2831-40. | selected |
| 179 | A prospective randomized trial of 0.010" versus 0.014" balloon PTCA systems and interventional fellow versus attending physician as primary operator in elective PTCA: economic, technical, and clinical end points. Talley JD, Mauldin PD, Leesar MA, Becker ER. J Interv Cardiol. 1995 Dec;8(6):623-32. | Excluded : other intervention was studied |
| 180 | Better functional status in American than Canadian patients with heart disease: an effect of medical care? Pilote L, Bourassa MG, Bacon C, Bost J, Detre K, Mark DB, Pitt B, Reeder G, Rogers WJ, Ryan T, et al. J Am Coll Cardiol. 1995 Nov 1;26(5):1115-20. | Excluded : other intervention was studied |
| 181 | In-hospital and one-year economic outcomes after coronary stenting or balloon angioplasty. Results from a randomized clinical trial. Stent Restenosis Study Investigators. Cohen DJ, Krumholz HM, Sukin CA, Ho KK, Siegrist RB, Cleman M, Heuser RR, Brinker JA, Moses JW, Savage MP, et al. Circulation. 1995 Nov 1;92(9):2480-7. | selected |
| 182 | Peripheral vascular complications in the Coronary Angioplasty Versus Excisional Atherectomy Trial (CAVEAT-I). Omoigui NA, Califf RM, Pieper K, Keeler G, O'Hanesian MA, Berdan LG, Mark DB, Talley JD, Topol EJ. J Am Coll Cardiol. 1995 Oct;26(4):922-30. | Excluded : other intervention was studied |
| 183 | Postoperative physical therapy after coronary artery bypass surgery. Johnson D, Kelm C, To T, Hurst T, Naik C, Gulka I, Thomson D, East K, Osachoff J, Mayers I. Am J Respir Crit Care Med. 1995 Sep;152(3):953-8. | Excluded : other intervention was studied |
| 184 | Baseline and 6-month costs of primary angioplasty therapy for acute myocardial infarction: results from the primary angioplasty registry. Mark DB, O'Neill WW, Brodie B, Ivanhoe R, Knopf W, Taylor G, O'Keefe JH, Grines CL, Davidson-Ray L, Knight JD, et al. J Am Coll Cardiol. 1995 Sep;26(3):688-95. | Excluded : follow up doesn't correspond to (less than 1 year or different than 1 and/or 3 years) |
| 185 | Predictors and sequelae of distal embolization during saphenous vein graft intervention from the CAVEAT-II trial. Coronary Angioplasty Versus Excisional Atherectomy Trial. Lefkovits J, Holmes DR, Califf RM, Safian RD, Pieper K, Keeler G, Topol EJ. Circulation. 1995 Aug 15;92(4):734-40. | Excluded : unconventional treatment |
| 186 | Oxygen cost of breathing for assisted spontaneous breathing modes: investigation into three states of pulmonary function. Weyland W, Schuhmann M, Rathgeber J, Weyland A, Fritz U, Laier-Groeneveld G, Schorn B, Braun U. Intensive Care Med. 1995 Mar;21(3):211-7. | Excluded : other intervention |
| 187 | Stress management for patients with heart disease: a pilot study. Turner L, Linden W, van der Wal R, Schamberger W. Heart Lung. 1995 Mar-Apr;24(2):145-53. | Excluded : other intervention |
| 188 | A single center randomized trial assessing use of a vascular hemostasis device vs. conventional manual compression following PTCA: what are the potential resource savings? Slaughter PM, Chetty R, Flintoft VF, Lewis S, Sykora K, Beattie DM, Schwartz L. Cathet Cardiovasc Diagn. 1995 Mar;34(3):210-4. | Excluded : other intervention |
| 189 | The impact of performing a clinical trial on patient outcomes: lessons from the Emory Angioplasty vs. Surgery Trial. King SB 3rd. Trans Am Clin Climatol Assoc. 1996;107:68-77 | it was a commentary |
| 190 | Use of the perfusion balloon as a primary dilation device. Phillips HR 3rd, Ohman EM, Labinaz M, Sketch MH Jr, Stack R. J Invasive Cardiol. 1995;7 Suppl B:17B-24B | Excluded : other intervention |
| 191 | Randomized prospective evaluation of prolonged versus abbreviated intravenous heparin therapy after coronary angioplasty. Friedman HZ, Cragg DR, Glazier SM, Gangadharan V, Marsalese DL, Schreiber TL, O'Neill WW. J Am Coll Cardiol. 1994 Nov 1;24(5):1214-9. | Excluded : other intervention |
| 192 | Health service costs of coronary angioplasty and coronary artery bypass surgery: the Randomised Intervention Treatment of Angina (RITA) trial. Sculpher MJ, Seed P, Henderson RA, Buxton MJ, Pocock SJ, Parker J, Joy MD, Sowton E, Hampton JR. Lancet. 1994 Oct 1;344(8927):927-30. | Excluded : follow up doesn't correspond to (less than 1 year or different than 1 and/or 3 years) |
| 193 | A comparison of balloon-expandable-stent implantation with balloon angioplasty in patients with coronary artery disease. Benestent Study Group. Serruys PW, de Jaegere P, Kiemeneij F, Macaya C, Rutsch W, Heyndrickx G, Emanuelsson H, Marco J, Legrand V, Materne P, et al. N Engl J Med. 1994 Aug 25;331(8):489-95. | Excluded : follow up doesn't correspond to (less than 1 year or different than 1 and/or 3 years) |
| 194 | Preliminary report of an ongoing phase I/II dose range, safety and efficacy study of iodine-123-phenylpentadecanoic acid for the identification of viable myocardium. Hansen CL. J Nucl Med. 1994 Apr;35(4 Suppl):38S-42S. | Excluded : other intervention |
| 195 | Ceftriaxone vs cefuroxime for infection prophylaxis in coronary bypass surgery. Sisto T, Laurikka J, Tarkka MR. Scand J Thorac Cardiovasc Surg. 1994;28(3-4):143-8. | Excluded : other intervention |
| 196 | Asymptomatic cardiac ischemia pilot (ACIP). Conti CR, Bourassa MG, Chaitman BR, Geller NL, Knatterud GL, Pepine CJ, Pratt C, Sopko G. Trans Am Clin Climatol Assoc. 1995;106:77-83 | Excluded : incomplete data (no economic data available) |
| 197 | Argentine randomized trial of percutaneous transluminal coronary angioplasty versus coronary artery bypass surgery in multivessel disease (ERACI): in-hospital results and 1-year follow-up. ERACI Group. Rodriguez A, Boullon F, Perez-Baliño N, Paviotti C, Liprandi MI, Palacios IF. J Am Coll Cardiol. 1993 Oct;22(4):1060-7. | selected |
| 198 | A comparison of directional atherectomy with coronary angioplasty in patients with coronary artery disease. The CAVEAT Study Group. Topol EJ, Leya F, Pinkerton CA, Whitlow PL, Hofling B, Simonton CA, Masden RR, Serruys PW, Leon MB, Williams DO, et al. N Engl J Med. 1993 Jul 22;329(4):221-7. | Excluded : other intervention |
| 199 | Comparison of angiographic center and local site analysis of PTCA results in a multicenter angioplasty-restenosis trial. The M Heart Group. Martinelli MJ, Deutsch E, Ferraro A, Bove AA. Cathet Cardiovasc Diagn. 1992 Sep;27(1):8-13. | Excluded : other intervention |
| 200 | Multicentre European trials: their design, execution and relevance. Hugenholtz PG, Lubsen J. Eur Heart J. 1989 Dec;10 Suppl H:92-100. | Excluded : it was a commentary |
| 201 | Long-term efficacy of coronary artery bypass surgery. Konttinen MP. Scand J Thorac Cardiovasc Surg. 1987;21(3):229-31. | Excluded : no randomization |
| **EMBASE** | | |
| 202 | A penny-per-ounce tax on sugar-sweetened beverages would cut health and cost burdens of diabetes Wang Y.C., Coxson P., Shen Y.-M., Goldman L., Bibbins-Domingo K. Health Affairs 2012 31:1 (199-207) | Excluded : other intervention |
| 203 | Concomitant carotid and cardiac disease: Short-term results of combined surgery in 76 patients Menegolo M., Frigatti P., Antonello M., Battocchio P., Ferretto L., Tarzia V., Rizzoli G., Gerosa G., Grego F. Italian Journal of Vascular and Endovascular Surgery 2010 17:4 (243-251) | Excluded : other intervention |
| 204 | Identification of asymptomatic type 2 diabetes mellitus patients with a low, intermediate and high risk of ischaemic heart disease: Is there an algorithm? Poulsen M.K., Henriksen J.E., Vach W., Dahl J., Møller J.E., Johansen A., Gerke O., Haghfelt T., Høilund-Carlsen P.F., Beck-Nielsen H. Diabetologia 2010 53:4 (659-667) | Excluded : other intervention |
| 205 | Predictors, barriers, and facilitators of lipid-lowering medication use among African Americans in a Primary Care Clinic Schwartz K.L., Dailey R., Bartoces M., Binienda J., Archer C., Neale A.V. Journal of the National Medical Association 2009 101:9 (944-952) | Excluded : other intervention |
| 206 | Fatal and nonfatal cardiovascular disease and the use of therapies for secondary prevention in a rural region of india Joshi R., Chow C.K., Raju P.K., Raju R., Reddy K.S., MacMahon S., Lopez A.D., Neal B. Circulation 2009 119:14 (1950-1955) | Excluded : other intervention |
| 207 | Coronary Heart Disease Attributable to Passive Smoking. CHD Policy Model Lightwood J.M., Coxson P.G., Bibbins-Domingo K., Williams L.W., Goldman L. American Journal of Preventive Medicine 2009 36:1 (13-20) | Excluded : other intervention |
| 208 | Practice characteristics and prescribing of cardiovascular drugs in areas with higher risk of CHD in Scotland: Cross-sectional study McLean G. International Journal for Equity in Health 2008 7 Article Number 18 | Excluded : other intervention |
| 209 | Simple adaptation of current abdominal aortic aneurysm screening programs may address all-cause cardiovascular mortality. Prospective observational cohort study Waterhouse D.F., Cahill R.A. American Heart Journal 2008 155:5 (938-945 | Excluded : other intervention |
| 210 | The cost-effectiveness of sibutramine in non-diabetic obese patients: Evidence from four Western countries Ara R., Brennan A. Obesity Reviews 2007 8:4 (363-371) | Excluded : other intervention |
| 211 | Cardiac resynchronisation therapy: What kind of equipment using? Leclercq C. Annales de Cardiologie et d'Angeiologie 2005 54:1 SPEC. ISS. (12-16) | Excluded : other intervention |
| 212 | One year after post-hospital rehabilitation - Which benefits remain? Laimer H. Journal fur Kardiologie 2004 11:11 (437-440) | Excluded : other intervention |
| 213 | Trends in fatal and non-fatal coronary heart disease events in Finland during 1991-2001 Pajunen P., Pääkkönen R., Juolevi A., Hämäläinen H., Keskimäki I., Laatikainen T., Moltchanov V., Niemi M., Rintanen H., Salomaa V. Scandinavian Cardiovascular Journal 2004 38:6 (340-344) | Excluded : no randomization |
| 214 | Results and cost of meeting the National Service Framework for Coronary Heart Disease requirement for 12 month follow-up after acute coronary events Evans J., Turner S., Bethell H. Journal of Public Health 2004 26:2 (185-186) | Excluded : population with different inclusion criteria (acute myocardial infarction) |
| 215 | Workload implications of identifying patients with ischaemic heart disease in primary care: Population-based study Gray J., Ekins M., Scammell A., Carroll K., Majeed A. Journal of Public Health Medicine 2003 25:3 (223-227) | Excluded : no randomization |
| 216 | Part III. Can we turn back the clock or modify the adverse dynamics? Programme and policy issues: Influencing public nutrition for non-communicable disease prevention: From community intervention to national programme - Experiences from Finland Pekka P., Pirjo P., Ulla U. Public Health Nutrition 2002 5:1 A (245-251) | Excluded : other intervention |
| 217 | Planning treatment of ischemic heart disease with partially observable Markov decision processes Hauskrecht M., Fraser H. Artificial Intelligence in Medicine 2000 18:3 (221-244) | Excluded : no randomization |
| 218 | Analysing and improving the diagnosis of ischaemic heart disease with machine learning Kukar M., Kononenko I., Grošelj C., Kralj K., Fettich J. Artificial Intelligence in Medicine 1999 16:1 (25-50) | Excluded : other intervention |
| 219 | Socioeconomic aspects of changing mortality from ischemic heart disease in Switzerland 1988-1993 Sagmeister M., Gessner U., Horisberger B., Gutzwiller F. Schweizerische Medizinische Wochenschrift 1998 128:10 (356-362) | Excluded : no randomization |
| 220 | New Zealand guidelines for the management of dyslipidaemia: Implications for treatment in an urban New Zealand population North D., Priest P., Lay-Yee R., Jackson R. New Zealand Medical Journal 1996 109:1020 (134-137) | Excluded : other intervention |
| 221 | Utilisation of the pre-operative ECG Callaghan L.C., Edwards N.D., Reilly C.S. Anaesthesia 1995 50:6 (488-490) | Excluded : other intervention |
| 222 | Cost effectiveness of work-site cholesterol screening and intervention programs Wilson M.G., Edmunson J., DeJoy D.M. Journal of Occupational Medicine 1992 34:6 (642-649) | Excluded : other intervention |
| 223 | An economic evaluation of lovastatin for cholesterol lowering and coronary artery disease reduction Hay J.W., Wittels E.H., Gotto Jr. A.M. American Journal of Cardiology 1991 67:9 (789-796) | Excluded : other intervention |
| 224 | Medical costs of coronary artery disease in the United States Wittels E.H., Hay J.W., Gotto Jr. A.M. American Journal of Cardiology 1990 65:7 (432-440) | Excluded : economic model |
| 225 | Late follow-up of 781 patients undergoing percutaneous transluminal coronary angioplasty or coronary artery bypass grafting for an isolated obstruction in the left anterior descending coronary artery Kramer J.R., Proudfit W.L., Loop F.D., Goormastic M., Zimmerman K., Simpfendorfer C., Horner G. American Heart Journal 1989 118:6 (1144-1153) | Excluded : no randomization |
| 226 | Issues regarding public screening for cholesterol levels in Australia James R., Tyler C., Van Beurden E. Australian Health Review 1989 12:1 (56-64) | Excluded : other intervention |
| 227 | A simple and economical equipment arrangement for percutaneous transluminal coronary angioplasty Haraphongse M., Rossall R.E. Canadian Journal of Cardiology 1989 5:5 (247-248) | Excluded : no randomization |
| 228 | Noninvasive and angiographic evaluation of coronary artery disease in patients with peripheral vascular disease Youngman D.J., Dove T., Boccuzzi S.J., Price H.L. American Journal of Cardiology 1989 63:20 (1446-1449) | Excluded : other intervention |
| 229 | Health family trees: A tool for finding and helping young family members of coronary and cancer prone pedigrees in Texas and Utah Williams R.R., Hunt S.C., Barlow G.K., Chamberlain R.M., Weinberg A.D., Cooper H.P., Carbonari J.P., Gotto Jr. A.M. American Journal of Public Health 1988 78:10 (1283-1286) | Excluded : other intervention |
| 230 | Effect of the Johnson & Johnson Live for Life program on employee smoking Shipley R.H., Orleans C.T., Wilbur C.S., Piserchia P.V., McFadden D.W. Preventive Medicine 1988 17:1 (25-34) | Excluded : other intervention |
| 231 | Ambulatory medical care for elderly diabetics: The fredericia survey of diabetic and fasting hyperglycaemic subjects aged 60-74 years Damsgaard E.M., Froland A., Holm N. Diabetic Medicine 1987 4:6 (534-538) | Excluded : other intervention |
| 232 | Benefits of experience. Treating coronary artery disease Hemenway D., Sherman H., Mudge Jr. G.H., et al. Medical Care 1986 24:2 (125-133) | Excluded : no randomization |
| 233 | Diagnostic testing for coronary artery disease in a large population Bloom B.S., Soper K.A. American Journal of Preventive Medicine 1986 2:1 (35-41) | Excluded : other intervention |
| 234 | Safety of outpatient cardiac catheterizations Klinke W.P., Kubac G., Talibi T., Lee S.J.K. American Journal of Cardiology 1985 56:10 (639-641) | Excluded : other intervention |
| 235 | Coronary angioplasty: Current concepts Coppinger Warren S., Warren S.G. American Family Physician 1985 32:2 (145-149) | Excluded : other intervention |
| **COCHRANE** | | |
| 236 | Clinical and economic impact of diabetes mellitus on percutaneous and surgical treatment of multivessel coronary disease patients: insights from the Arterial Revascularization Therapy Study (ARTS) trial. Abizaid A , Costa MA , Centemero M , Abizaid AS , Legrand VM , Limet RV , Schuler G , Mohr FW , Lindeboom W , Sousa AG , Sousa JE , vanHout B , Hugenholtz PG , Unger F , Serruys PW and Arterial Revascularization Therapy Study Group Circulation, 2001, 104(5), 533 | Excluded : doubloon |
| 237 | [Economic evaluation of different treatment strategies in patients with stable angina pectoris or asymptomatic myocardial ischemia on basis of results from the Asymptomatic-Cardiac-Ischemia Pilot study (ACIP)]. Szucs TD , Schwenkglenks M and Rutishauser W Medizinische Klinik (Munich, Germany : 1983), 2000, 95(1), 1 | Excluded : article in German |
| 238 | Prophylactic coronary artery revascularization for elective vascular surgery: study design. Veterans Affairs Cooperative Study Group on Coronary Artery Revascularization Prophylaxis for Elective Vascular Surgery. McFalls EO , Ward HB , Krupski WC , Goldman S , Littooy F , Eagle K , Nyman JA , Moritz T , McNabb S and Henderson WG Controlled clinical trials, 1999, 20(3), 297 | Excluded : doubloon |
| 239 | Arterial Revascularization Therapies Study (ARTS). Indian heart journal, 2001, 53(2), 239 | Excluded : doubloon |
| 240 | Drug eluting stents in Belgium: Health Technology Assessment (Structured abstract) M Neyt, H Van Brabandt, S Devriese, J Mahieu, A De Ridder, D De Graeve, C De Laet | Excluded : it was a review |
| 241 | New coronary revascularisation techniques - horizon scanning review (Structured abstract) National Horizon Scanning Centre 2001 | Excluded : it was a review |
| 242 | Transmyocardial revascularisation by laser (systematic review, expert panel) (Structured abstract) M-S Pauchard-Civadier, S Baffert, A-F Fay, N Jakobi-Rodrigues 1998 | Excluded : it was a review |
| 243 | Cost effectiveness of abciximab during routine medical practice (Structured abstract) Reed S O , Mullins C D and Magder L S Pharmacoeconomics, 2000, 18(3), 265-274 | Excluded : retrospective |
| 244 | Acute and long-term cost implications of coronary stenting (Structured abstract) Peterson E D , Cowper P A , DeLong E R , Zidar J P , Stack R S and Mark D B Journal of the American College of Cardiology, 1999, 33(6), 1610-1618 | Excluded : follow up doesn't correspond to (less than 1 year or different than 1 and/or 3 years) |
| 245 | Cost-effective selection of patients for coronary angiography (Structured abstract) Maddahi J and Gambhir S S Journal of Nuclear Cardiology, 1997, 4(2), S141-S151 | Excluded : other intervention |
| 246 | Incremental benefit and cost-effectiveness of high-dose statin therapy in high-risk patients with coronary artery disease (Structured abstract) Chan P S , Nallamothu B K , Gurm H S , Hayward R A and Vijan S Circulation, 2007, 115(18), 2398-2409 | Excluded : economic model |

**Further publications identified from searching reference list** (thanks to the references of selected, index summary of different journals of Cardiology, the websites of European and American societies of cardiology articles, etc.)

1. Cost-effectiveness of coronary angioplasty versus medical treatment: the impact of cost-shifting. Kinlay S. Aust N Z J Med. 1996;26(1):20-6.

2. Cost effectiveness of paclitaxel-eluting stents for patients undergoing percutaneous coronary revascularization: results from the TAXUS-IV Trial. Bakhai A, Stone GW, Mahoney E, Lavelle TA, Shi C, Berezin RH, Lahue BJ, Clark MA, Lacey MJ, Russell ME, Ellis SG, Hermiller JB, Cox DA, Cohen DJ; TAXUS-IV Investigators. J Am Coll Cardiol. 2006;48(2):253-61

3. One year cost effectiveness of sirolimus eluting stents compared with bare metal stents in the treatment of single native de novo coronary lesions: an analysis from the RAVEL trial.

van Hout BA, Serruys PW, Lemos PA, van den Brand MJ, van Es GA, Lindeboom WK, Morice MC. Heart. 2005;91(4):507-12.

4. Clinical Trial Update. ENDEAVOR I & II clinical program : long term follow-up. Meredith I and Wijns W. 4 September 2005, ESC Stockholm 2005

5. ENDEAVOR clinical program update: ENDEAVOR I: 4-year clinical follow-up. ENDEAVOR II: 3-year clinical follow-up. Zeiher A. May 22 2007, EuroPCR, Barcelona 2007
